# Confined Catalysis of Enzymes: Enhancement Strategies, Materials, and Biosensor Applications

**DOI:** 10.3390/jfb17080407

**Published:** 2026-08-15

**Authors:** Lijiao Zhang, Junyi Lu, Chenjie Yan, Wenjing Wang, Dajing Chen

**Affiliations:** Zhejiang Provincial Key Laboratory of Anti-Cancer Chinese Medicines and Natural Medicines, School of Pharmacy, Hangzhou Normal University, Hangzhou 311121, China; 2024112025070@stu.hznu.edu.cn (L.Z.); 2025112025115@stu.hznu.edu.cn (J.L.); 2025112025013@stu.hznu.edu.cn (C.Y.); 2024211505052@stu.hznu.edu.cn (W.W.)

**Keywords:** confined catalysis, immobilization, microenvironment, confined materials, biosensors

## Abstract

Confined catalysis enhances enzyme activity, stability, and selectivity by encapsulating enzymes in well-defined micro- or nanoscale environments. Unlike conventional immobilization strategies, which primarily stabilize enzymes, this approach actively regulates enzyme behavior, substrate transport, and product removal, addressing challenges in real-world biosensing. In this work, we connect confined catalysis principles with biosensor design requirements, emphasizing tailored microenvironments, hierarchical pore architectures, and cascade reaction optimization to improve sensitivity, selectivity, and operational stability. The review also highlights advanced strategies such as molecular imprinting, aptamer recognition, and DNA scaffold-guided co-localization, which enable precise substrate discrimination and efficient signal amplification. By integrating these confinement strategies with emerging materials, including biological macromolecular materials, amorphous porous materials, and crystalline framework materials, this work outlines pathways to next-generation biosensors with enhanced performance and practical applicability, addressing limitations overlooked in previous studies.

## 1. Introduction

### 1.1. The Application of Enzymes in Biosensing

Enzymes are highly efficient biocatalysts. They exhibit specific substrate recognition, operation under mild conditions, and environmental compatibility [1,2,3,4]. These properties make enzymes particularly attractive for biosensing applications, where catalytic amplification and molecular recognition are essential for achieving high sensitivity and selectivity [5]. Despite these benefits, enzyme instability poses a major limitation for practical use. Enzymatic activity depends on maintaining a well-defined three-dimensional structure, but enzymes are prone to conformational changes during storage, processing, or exposure to harsh environments. In addition, enzyme leaching during operation also limits reusability and long-term stability, increasing operational costs and hindering large-scale implementation.

Enzyme immobilization has emerged as an effective strategy to address these challenges. By anchoring enzymes onto suitable supports, immobilization can improve structural stability, suppress enzyme leakage, extend operational lifetime, and reduce assay costs [6]. However, maintaining or enhancing catalytic activity after immobilization remains a critical challenge. The development of advanced immobilization materials and strategies capable of preserving or improving enzymatic function continues to be a major research focus in biosensing [7].

### 1.2. Advantages of Confinement Catalysis

**Figure 1:**
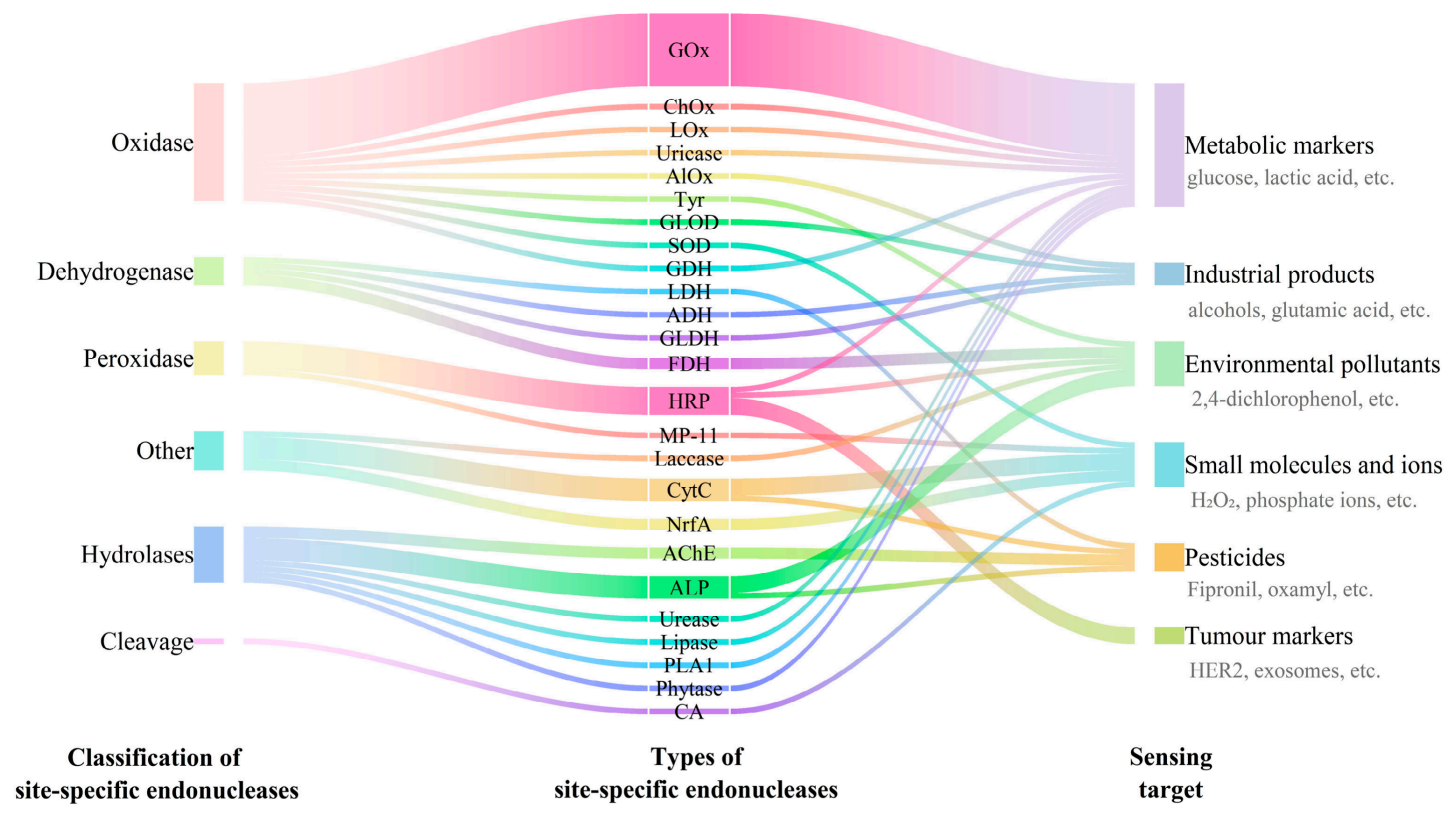
Types of confined catalytic enzymes and sensing targets.

Addressing the limitations of conventional enzyme immobilization, confined catalysis has gained increasing attention due to its spatial confinement effects and tunable microenvironments. This strategy involves embedding enzyme molecules within defined micro- or nano-scale spaces, where their mobility is restricted and reactions occur in a controlled manner [8,9]. Such confinement not only modulates catalytic activity and substrate selectivity but also helps maintain the three-dimensional structure of the enzymes. However, the advantages of confinement do not simply increase with enhanced spatial restriction. Excessive confinement may limit enzyme flexibility, hinder substrate diffusion, and cause product accumulation. Therefore, the key challenge is to establish an optimal confinement environment that balances structural stabilization, molecular transport, and catalytic accessibility. Today, confined catalysis has been widely applied in the development of enzyme-based biosensors [10,11,12,13,14,15] and is extensively used for the rapid detection of various substances (Figure 1), such as disease biomarkers and environmental pollutants, providing efficient and reliable tools for biosensing [16,17].

In addition to natural enzymes, confined microenvironments can improve the performance of nanozymes, which inherently mimic enzymatic activity. Although nanozymes offer high structural stability and potential for biosensor applications, their catalytic efficiency and substrate specificity are generally lower than those of natural enzymes. Confinement within micro- or nano-scale environments has been shown to enhance the catalytic activity and interference resistance of certain nanozymes [18,19].

The development of confined catalysis has therefore expanded the conceptual framework of enzyme immobilization. Rather than serving solely as passive supports, immobilization matrices are increasingly designed as functional catalytic microenvironments that actively regulate enzyme behavior. This shift from random immobilization to rational microenvironment engineering provides new opportunities for constructing high performance enzyme-based biosensors.

### 1.3. Enzyme-Confinement Catalytic Materials

The concept of confined catalysis is inspired by biological systems. Living organisms achieve highly efficient biochemical transformations through hierarchical confinement, ranging from cellular compartments and organelles to the precisely structured active sites of enzymes. These naturally evolved confined environments regulate substrate transport, concentrate reactants, and maintain optimal catalytic conditions, thereby enabling exceptional catalytic efficiency.

Inspired by these biological architectures, a variety of artificial confinement materials have been developed. Biopolymer-based assemblies [20,21,22,23] and amorphous porous materials [24,25,26] are widely used to improve enzyme stability and catalytic performance. With advances in nanotechnology, the emergence of crystalline framework materials with tunable pore structures such as metal–organic frameworks [27] and covalent organic frameworks [28,29,30] has also provided ideal confined spaces for enzyme molecules. Owing to their tunable pore size, high surface area, and versatile chemical functionality, these materials provide well-defined confined spaces that are highly suitable for accommodating enzyme molecules [31,32].

### 1.4. Advances in Confined Enzyme Catalysis

In recent years, several reviews have summarized the development of confined catalysis from different perspectives, covering the design of confinement materials, the structure–activity relationships of enzyme biocomposites, and the influence of confined microenvironments on catalytic performance. These studies have highlighted the roles of porous organic frameworks and metal–organic frameworks (MOFs) in enhancing enzyme stability, regulating catalytic activity and selectivity, and expanding applications in fields such as biocatalysis and biomedicine [33,34,35]. However, existing reviews have mainly emphasized specific classes of confinement materials or individual catalytic mechanisms, whereas a systematic understanding of how material structure, surface properties, and confined microenvironments collectively govern enzyme performance remains lacking.

Accordingly, this review highlights recent advances in enhancing confined enzyme catalytic performance through material modification and structural regulation. Existing material systems for confined enzyme catalysis are systematically classified based on their structural characteristics, with detailed analysis of their structural features, confinement mechanisms, and regulatory effects on enzymatic catalysis. Furthermore, this review focuses on biosensing applications and explores the synergistic integration of confinement materials with enzyme catalytic regulation strategies. This review aims to illustrate the intrinsic relationships among confinement material structures, microenvironment regulation, and enzymatic performance, while systematically uncovering how material properties govern the optimization of confined enzyme systems. These insights provide valuable guidance for the rational design and biosensing applications of high-performance confined enzyme platforms.

## 2. Strategies for Enhancing the Catalytic Performance of Confined Enzymes

Spatial confinement of enzymes can profoundly influence enzyme–substrate interactions, local microenvironments, and encapsulation effects, often enhancing catalytic efficiency, substrate specificity, and stability relative to conventional immobilization methods. The performance of confined enzymes is determined by multiple factors, including carrier pore architecture, shell thickness, surface chemistry, and enzyme–carrier interactions. This section systematically reviews strategies for optimizing enzymatic performance within confined microenvironments.

### 2.1. Strategies for Enhancing the Catalytic Efficiency of Confined Enzymes

**Figure 2:**
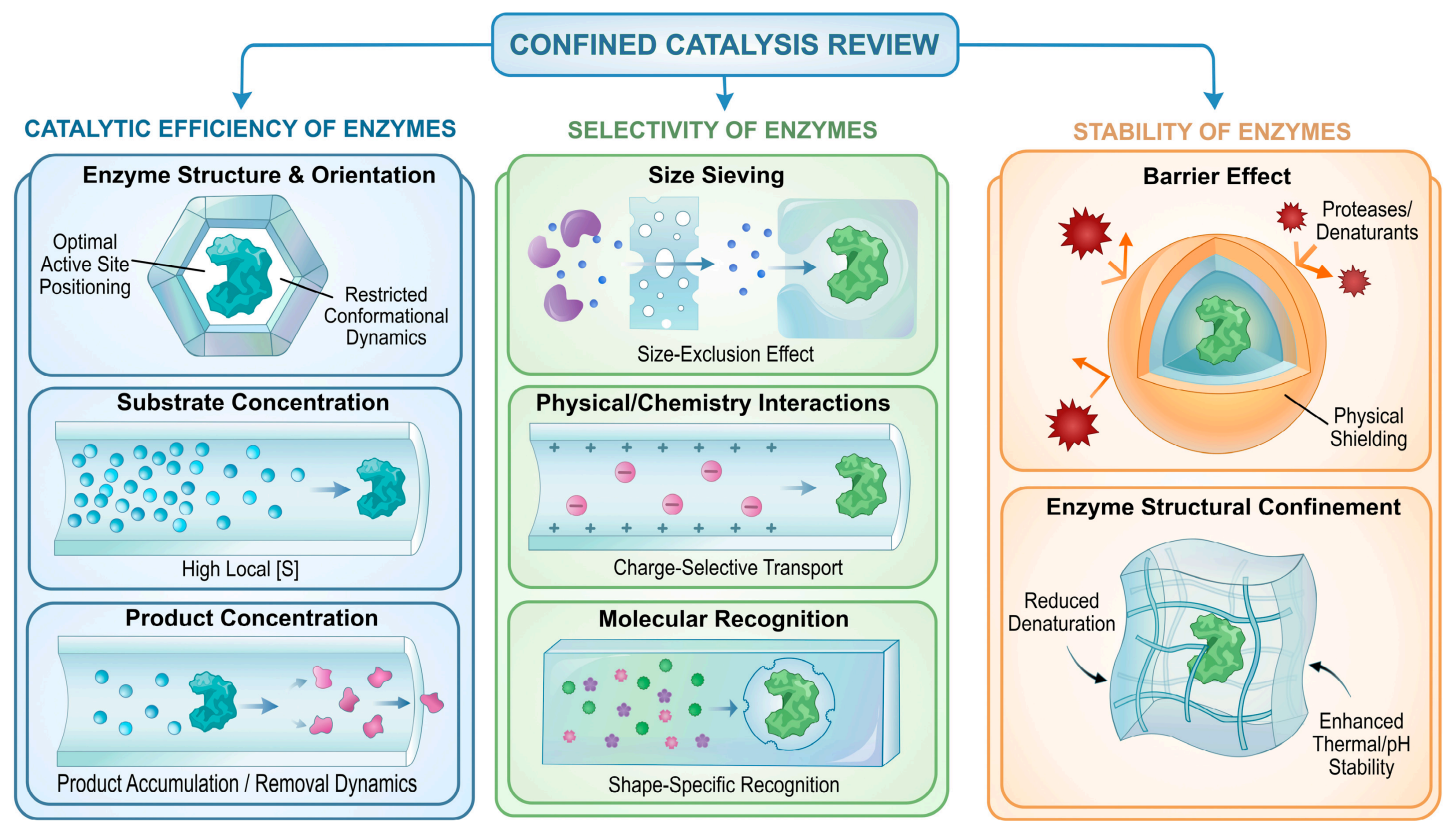
Summary of Strategies for Enhancing Confined Catalytic Performance.

This section reviews approaches for enhancing the catalytic efficiency of enzymes in confined spaces, focusing on the interaction among enzymes, substrates, and products (Figure 2). Optimizing each element is essential for maximizing performance.

#### 2.1.1. Enzyme Conformation and Orientation

The catalytic activity of an enzyme is inherently determined by its conformational state, making precise regulation of enzyme conformation a key strategy for enhancing catalytic efficiency. Within confined microenvironments, nano–bio interfacial interactions between enzymes and the pore walls of carriers can induce conformational changes, thereby altering catalytic performance. For example, a study immobilized formaldehyde dehydrogenase (FDH) on mesoporous silica and showed that when the pore size was 12.3 nm, closely matching the dimensions of the enzyme (8.6 nm × 8.6 nm × 19.0 nm), the confined space created an ideal environment, resulting in maximal FDH activity. Furthermore, phenyl-functionalization of the pores reduced the Michaelis constant from 2.84 mM to 0.43 mM, bringing catalytic efficiency close to that of the native enzyme, with a corresponding increase in enzyme activity. This enhancement was attributed to hydrophobic interactions between the phenyl groups and hydrophobic regions of the enzyme, which stabilized the enzyme conformation and facilitated exposure of the active site [36]. These results indicated that functionalizing material pores to finely tune enzyme–carrier interfacial interactions is an effective strategy to modify enzyme conformation and enhance catalytic performance [37].

**Figure 3:**
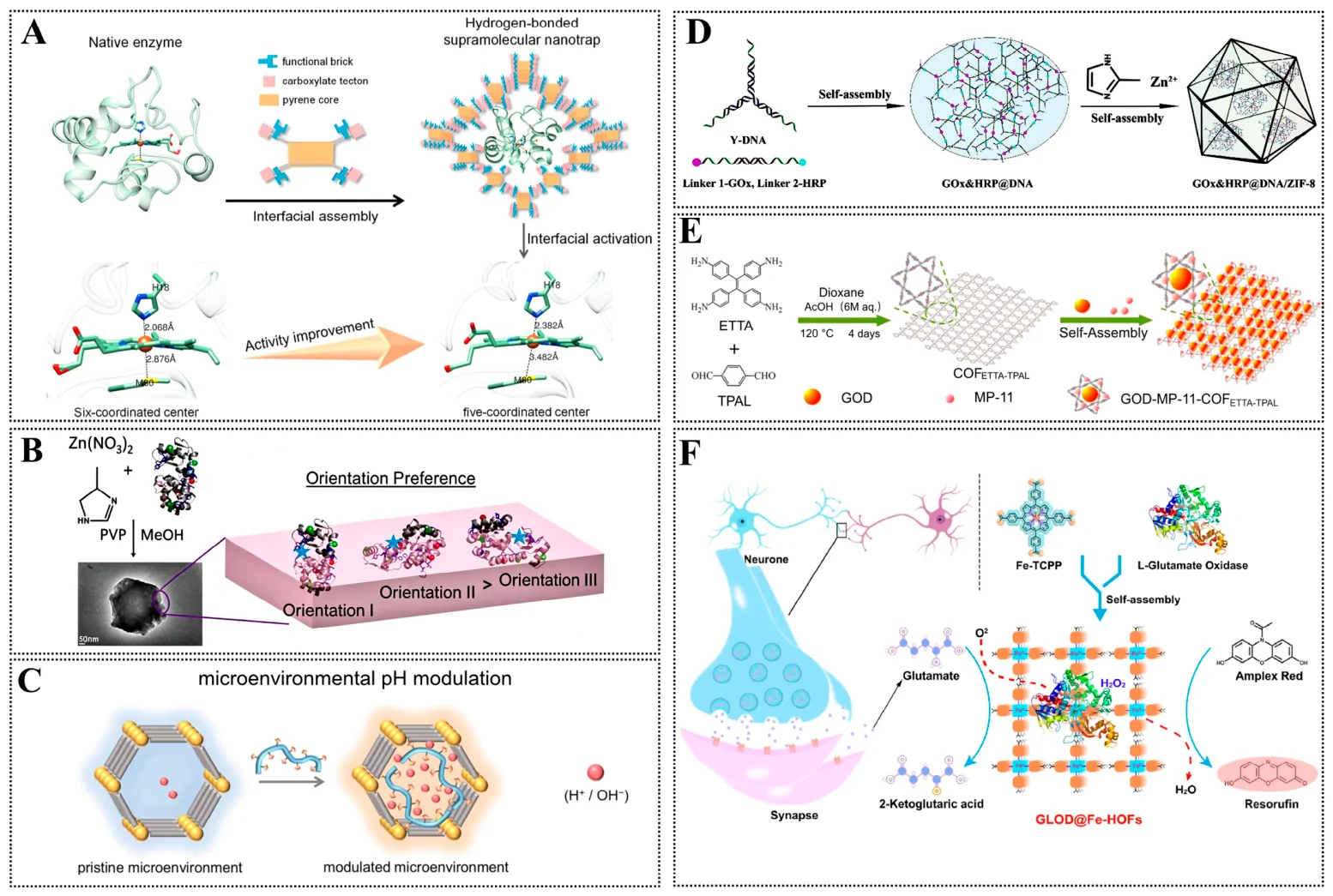
Representative strategies for enhancing confined enzyme catalysis. (**A**) Synergistic Interface Activation by Hydrogen-Bonded Supramolecular Nanotraps and Hydrophobic Groups. Reprinted with permission from Ref. [39]. Copyright 2023 American Chemical Society. (**B**) Orientation distribution of T4L on ZIF-8, with the star indicating the enzyme active site. Reprinted with permission from Ref. [42]. Copyright 2018 American Chemical Society. (**C**) pH modulation of the microenvironment via polymers. Reprinted from Ref. [60]. (**D**) Co-localization of cascade enzymes via DNA tetrahedra facilitates catalysis. Reprinted with permission from Ref. [67]. Copyright 2019 Elsevier B.V. (**E**) Spatial separation of different enzymes in sub-compartments enables ordered cascade catalysis. Reprinted with permission from Ref. [70]. Copyright 2019 Elsevier B.V. (**F**) Fe-HOF functional material with L-glutamate oxidase enhances cascade efficiency. Reprinted with permission from Ref. [74]. Copyright 2024 Elsevier B.V.

Based on this principle, an increasing number of studies have employed pore engineering in porous matrices to modulate the local chemical microenvironment. Taking cytochrome c (Cyt c) as an example, Xing et al. adjusted the density of epoxy functional groups within the channels of covalent organic frameworks (COFs) to finely tune covalent interactions between the enzyme and carrier, which effectively reduced the α-helical content of Cyt c and exposed its heme active center, resulting in immobilized Cyt c activity approximately six times that of the free enzyme [38]. Huang et al. (Figure 3A) introduced methyl groups into Hydrogen-bonded Organic Frameworks (HOFs) channels, where combined hydrogen-bonding and hydrophobic interactions shifted the Cyt c active center from a low-activity six-coordinate state to a substrate-favorable five-coordinate configuration, increasing the reaction rate by 4.4-fold [39]. Together with COF-based pore functionalization, these studies showed that tuning enzyme–carrier interactions can enhance catalysis by regulating enzyme conformation. Numerous subsequent studies have similarly adopted this strategy to control enzyme conformation, demonstrating its general applicability in improving enzymatic performance [31,40,41]. However, the key point of confinement-activated enzymes lies in achieving a delicate balance between stabilization and conformational flexibility. While moderate interfacial interactions can stabilize catalytically favorable conformations, excessive interactions may lock the enzyme into non-native states and reduce its activity. Therefore, an optimal balance must be carefully maintained. In addition, defect engineering, such as enlarging pores or reducing crystallinity, can also improve enzyme conformation while maintaining stability and substrate specificity [17,27].

Besides conformation, enzyme orientation within confined spaces also affects substrate accessibility. Generally, enzymes with active sites oriented toward the solvent exhibit higher catalytic activity. For example, Pan et al. demonstrated that the orientation of lysozyme on metal–organic frameworks (MOFs) was guided by π–π interactions between aromatic residues and imidazole ligands (Figure 3B) [42], which positioned the active site toward the solvent and enhanced substrate access. In addition, studies showed that surface functionalization, particularly with thiol groups, could further regulate enzyme orientation and improve catalytic performance [43]. A recent study grafted carboxymethyl-β-cyclodextrin (CD) onto large-pore UiO-66-NH_2_, combining dynamic hydrogen bonding with MOF-imposed spatial constraints to regulate enzyme orientation. Density functional theory calculations indicated that CD modification reduced substrate adsorption energy from 593.36 kJ·mol^−1^ to −76.40 kJ·mol^−1^, demonstrating enhanced substrate affinity [44].

Overall, carrier surface properties including hydrophobicity, charge, and functional group distribution serve as critical determinants of immobilized enzyme performance. Through targeted surface modification or structural engineering, enzymes can be positioned and stabilized in catalytically favorable states.

#### 2.1.2. Substrate Accessibility and Enrichment

Substrate concentration is a decisive factor in enzyme catalytic efficiency. In confined microenvironments, substrate transport to enzyme active sites is often limited, resulting in a lowered catalytic performance. This limitation can be addressed through rational design of confined carriers: (i) optimizing pore architectures to minimize mass transfer resistance, and (ii) enhancing carrier–substrate interactions to elevate local substrate concentration.

Diffusion constraints typically arise from pores that are either too small or overly dense. Enlarging pore sizes [45,46,47] or increasing porosity [48] can improve substrate transport efficiency; nevertheless, simply increasing pore volume is not an ideal solution because weakened confinement can accelerate enzyme leakage and reduce local substrate enrichment. Hierarchical pore structures therefore represent a more rational strategy by simultaneously providing enzyme accommodation, substrate diffusion pathways, and retention capability. To balance these factors, hierarchical pore architectures, featuring large cavities for enzyme accommodation and smaller mesoporous channels for substrate diffusion, have been developed to preserve confinement while enhancing mass transfer [49]. For example, Lykourinou et al. encapsulated MP-11 within Tb-mesoMOF featuring 3.9/4.7 nm nanocages and 3.0/4.1 nm mesoporous channels. The large cages accommodated the enzyme, while the smaller pores allowed substrate diffusion, which resulted in a catalytic conversion rate of 48.7%, significantly higher than that of conventional mesoporous silica materials (17.0%). Moreover, the activity remained stable after six cycles, whereas in the MCM-41 system, enzyme leakage led to only 28% activity left after three cycles, highlighting the unique advantage of hierarchical pore structures in balancing mass transfer and confinement [50].

For biosensors targeting low-concentration analytes, substrate enrichment near active sites can further enhance catalytic efficiency. This can be achieved via electrostatic [51,52], hydrophobic [32], and coordination interactions [53]. Electrostatic interactions are most effective for charged analytes, such as metal ions or ionic metabolites; hydrophobic interactions are more suitable for nonpolar or moderately hydrophobic molecules, including many small-molecule drugs or organic pollutants. Coordination interactions are particularly appropriate for analytes capable of forming specific coordination bonds, such as transition metal ions. Selecting the most suitable interaction type according to the chemical properties of the target analyte can effectively enhance substrate enrichment. Gil-Garcia, M. et al. showed that negatively charged substrates NADH and cofactor FAD were enriched in highly cationic Dbp1-NOX assemblies [52]. Guo et al. showed that hydrophobic COF layers increased the local concentration of benzaldehyde within pores [32]. This strategy is particularly effective in complex ionic media or for poorly soluble substrates, providing a route to high sensitivity, low-detection-limit biosensors. However, excessive substrate enrichment may also induce non-specific adsorption and substrate inhibition, particularly in complex biological matrices containing abundant competing molecules. Therefore, substrate enrichment could be considered in combination with selective transport mechanisms to avoid compromising analytical specificity.

Additionally, enzyme encapsulation within water-containing microporous substrate offers another strategy. Water clusters increase pore hydrophilicity and, through steric effects, reduce unfavorable substrate–pore wall interactions, preventing channel blockage and facilitating diffusion. In Zn-BTC MOF, one-dimensional water clusters within the pores enhanced the catalytic activity of encapsulated cytochrome c and lipase (PS) and surpassed that of the free enzymes. Molecular dynamics simulations confirmed that water molecules accelerated substrate diffusion by weakening the hydrogen bonding between H_2_O_2_ and the carboxyl groups on the pore walls [54].

In summary, by modulating the pore structure and surface chemistry of confined carriers, substrate transport to the enzyme active sites can be enhanced, effectively alleviating diffusion limitations potentially caused by confined catalysis, thereby significantly improving enzymatic catalytic efficiency.

#### 2.1.3. Product Accumulation and Microenvironment Regulation

In confined microenvironments, enzymatic reaction products diffuse more slowly than in bulk solution, leading to local accumulation at the nanoscale that can significantly affect enzyme activity [55]. Among these products, protons and toxic byproducts are particularly influential.

Protons, one of the most prevalent enzymatic products, can cause pronounced local pH fluctuations. For instance, oxidases (e.g., glucose oxidase [56]) release protons during catalysis, thereby lowering the local pH. In contrast, urease consumes protons, leading to an increase in local pH [57]. Unlike conventional enzyme systems where bulk solution conditions dominate reaction behavior, confined systems generate heterogeneous microenvironments with spatially varying proton concentration and intermediate distribution. This feature provides new opportunities for catalytic regulation but also introduces additional complexity in predicting enzyme kinetics. Since most enzymes are highly sensitive to pH, their activity is significantly enhanced when the local pH approaches the optimal range. Therefore, precise control of proton distribution in confined microenvironments is a key strategy to maintain or enhance enzyme activity. For example, urease encapsulated in liposomal nanoreactors raised the internal pH by consuming protons, creating a positive feedback loop that boosted enzyme activity. Adjusting the membrane thickness (20–30 nm) and surface charge allowed precise tuning of proton replenishment, providing fine control over reaction kinetics and catalytic efficiency [58].

The proton-accepting or -releasing ability of the carrier can significantly modulate the local pH and thus influence enzyme activity. Zhang et al. immobilized glucose oxidase (GOx) and horseradish peroxidase (HRP) on a polyanionic carrier that reduced the local pH from 7.5 to 5.5, increasing their activities by 1.2-fold and 4-fold, respectively. They further found that the high-density negative charges of the DNA scaffold attracted protons electrostatically, lowering the local pH by 1–2 units relative to bulk solution and markedly enhancing enzyme activity [59]. This demonstrates that the carrier’s intrinsic pH-modulating capacity can buffer pH changes, maintaining the microenvironment within the optimal range for catalysis.

When the carrier alone cannot sufficiently alter pH, pH-responsive polymers or gold nanoparticles can be incorporated into the pores. For example, Li et al. confined polyacrylic acid (PAA) within MOF nanopores; the carboxyl groups of PAA acted as a proton reservoir, locally releasing protons to lower the pH around the active sites, enabling high catalytic activity even under neutral bulk conditions (Figure 3C) [60]. Similarly, confining the basic polymer polyethyleneimine (PEI) in Zr-MOF increased the local pH, enhancing MOF hydrolytic enzyme-like activity. Likewise, cationic gold nanoparticles (GNPs) can electrostatically enrich carbonate ions, amplifying the local pH increase induced by urease and further improving enzyme performance [57]. These studies indicated that employing pH-responsive carriers or functionalized carriers to manipulate protons within confined environments is an effective strategy for enhancing enzyme activity.

In addition to protons, the accumulation of toxic byproducts can impair enzyme function through mechanisms such as conformational distortion, active site binding, or oxidative stress. Timely removal of these byproducts is therefore crucial. One approach is enzymatic conversion into harmless products, such as HRP-mediated decomposition of H_2_O_2_ generated during GOx-catalyzed glucose oxidation. Another strategy is to accelerate byproduct efflux, either by enlarging carrier pores to facilitate diffusion or by minimizing interactions between the byproducts and the confined carrier.

In conclusion, dynamic regulation of microenvironmental pH serves as an effective strategy to modulate enzyme activity. Coupling this with the unique proton dynamics of confined systems and efficient removal of toxic byproducts offers a promising approach to significantly enhance the catalytic efficiency of confined enzyme systems.

#### 2.1.4. Synergistic Effects in Confined Microenvironments

One key advantage of confined catalysis is its ability to facilitate highly efficient cascade reactions through molecular synergy. This feature is particularly valuable in amplifying biosensor signals. For example, dual-enzyme cascade systems, such as glucose oxidase–horseradish peroxidase (GOx-HRP) for glucose detection [61,62] and lactate dehydrogenase–horseradish peroxidase (LDH-HRP) for lactate detection [63], can both enhance detection sensitivity. In addition, triple-enzyme systems, such as cholinesterase-cholesterol oxidase–horseradish peroxidase (ChE-ChOx-HRP) for cholesterol detection [64] and alkaline phosphatase–tyrosinase–horseradish peroxidase (ALP-TYR-HRP) for the cardiac injury biomarker cardiac Troponin I [65], can achieve higher sensitivity and quantitative capability.

In multi-enzyme co-confined systems, substrate transport within the confined space strongly influences cascade efficiency. Establishing substrate channeling is therefore an effective strategy to enhance performance, typically achieved by reducing the distance between paired enzymes. However, while decreased enzyme spacing can facilitate intermediate transfer, the shortest distance does not necessarily result in maximal catalytic efficiency. Excessive enzyme crowding may compromise substrate accessibility, induce steric constraints, and perturb the intrinsic dynamics of individual enzymes. Therefore, optimal spatial organization rather than maximal proximity should be considered in cascade design. Physical proximity can be directly controlled through the rational design of confined carriers. For example, in the GOx–HRP system encapsulated within one-dimensional channels of HOFs, the close spatial arrangement reduced the diffusion distance of H_2_O_2_ intermediates to the nanoscale, leading to near-instantaneous transfer and a 4.25-fold increase in catalytic efficiency compared to the free enzymes [66]. Programmable DNA scaffolds enabled precise enzyme co-localization, minimizing intermediate diffusion and enhancing cascade efficiency. A Y-shaped scaffold co-localized GOx and HRP within ~30 base pairs, which increased substrate affinity 3.7-fold and catalytic efficiency 9.9-fold (Figure 3D) [67], illustrating the potential of combining DNA nanotechnology with confined carriers for efficient multi-enzyme biosensors. In the TRAP protein scaffold system, formate dehydrogenase 1 (FDH1) and alanine dehydrogenase 3 (AlaDH3) are co-localized within a distance of 3.5 nm, whereas the average distance between free enzymes at the same concentration is approximately 209 nm. This nanoscale spatial organization creates an auxiliary substrate channeling effect, resulting in a specific productivity of 5.21 g·genzyme^−1^·h^−1^ for the bienzyme system, which is 5-fold higher than that of the free enzyme mixture [68]. In another example, in the ZIF-8 confined system, ADH, LDH, and NAD^+^-loaded mesoporous silica nanoparticles (MSN) are co-encapsulated. The ZIF-8 shell restricts all components within a nanoscale volume, shortening the effective diffusion distance of the NADH intermediate between the enzymes and enabling in situ cofactor regeneration, thus achieving a 2.82-fold higher rate of lactate synthesis than that of the free enzymes [69].

Confined carriers can also achieve ordered multi-enzyme assembly through hierarchical structural design. In one example, a bimetallic covalent organic framework (COFETTA-TPAL) was used to construct a ratiometric electrochemical biosensor for glucose. Its dual pore sizes (3.06 nm and 0.87 nm) matched the dimensions of glucose oxidase (GOD) and microperoxidase-11 (MP-11), respectively, and directional immobilization via pore encapsulation and hydrogen bonding ensured orderly cascade progression (Figure 3E) [70]. In another example, superoxide dismutase 1 (SOD1) and cytochrome c (cyt c) were sequentially encapsulated into the nanoscale cavities of coordination cages, and these enzyme-loaded cages were further self-assembled into isomorphous single crystals with regular arrangement, constructing a hierarchical confined structure. Within this confined microenvironment, the co-encapsulated system catalyzed a cascade oxidation reaction at a rate 4.2-fold higher than that of a mixture of two separate crystals each immobilizing a single enzyme. This strategy achieves spatially controllable arrangement of enzyme active sites through hierarchical confinement, providing a new approach for developing structurally uniform, highly efficient, and stable multienzyme cascade catalytic systems [71].

Integrating catalytic functionality within the carrier can further streamline substrate transfer. Materials with intrinsic peroxidase-like activity allow cascade reactions to proceed without additional enzymes. For instance, GOx confined in a Co-MOF leveraged the MOF’s peroxidase-like activity to directly convert H_2_O_2_ generated by GOx, avoiding spatial compatibility issues and simplifying the system [72]. Similarly, Lu et al. and Ye et al. encapsulated glucose oxidase and glutamate oxidase (GLOD) within Fe-COF and Fe-HOF (Figure 3F) frameworks, respectively. Both studies utilized the iron centers within the porous frameworks as peroxidase-mimetic sites for near-field cascade reactions [73,74]. The Fe-COF@GOx system enabled linear glucose detection over a 10–1000 μM range with a detection limit of 1.4 μM, while Fe-HOF@GLOD exhibited linear glutamate detection from 0.05 to 5 μM with a detection limit of 0.02 μM, facilitating ultrasensitive measurements in mouse cerebrospinal fluid. Geng and colleagues confined alcohol oxidase (AOx) and aldehyde dehydrogenase (ALDH) inside a mesoporous MOF (MIL-101(Fe)) that has intrinsic catalase-like activity. In this nanoreactor, AOx generates H_2_O_2_ when oxidizing alcohol; the MOF quickly decomposes H_2_O_2_ into O_2_, which fuels AOx in a self-accelerating cascade. The confined space also ensures that the toxic aldehyde intermediate is immediately consumed by the adjacent ALDH. This NAD^+^-free cascade achieves an alcohol degradation rate > 0.5 mg·mL^−1^·h^−1^ with almost no aldehyde accumulation, showing that integrating catalytic functions into a porous carrier greatly enhances cascade efficiency [75].

In summary, cascade reactions represent one of the most prominent advantages of confined catalysis. By constraining multistep enzymatic reactions within nanoscale spaces, the product of one enzyme can be rapidly converted into the substrate of the subsequent enzyme, significantly reducing diffusion distances. Through rational design of confined space, the efficiency of cascade enzymatic catalysis and the overall reaction rate can be substantially enhanced.

### 2.2. Strategies for Enhancing the Stability of Site-Specific Enzymes

In confined catalytic systems, the operational stability and long-term usability of enzymes are key determinants for translating laboratory-scale concepts into practical applications. Confinement typically improves enzyme stability through two pathways [76]. First, the structural rigidity of the support material provides a physical barrier that protects enzymes from harsh external conditions [77]. Second, precise spatial arrangement imposes conformational constraints on enzyme molecules, which maintains the native conformation during catalysis [78]. Despite these advantages, the practical application of confined enzymatic systems is still hindered by carrier instability under harsh conditions and persistent enzyme leakage, both of which compromise long-term catalytic performance and durability.

#### 2.2.1. Enhancing Carrier Stability to Protect the Enzyme

In confined catalytic systems, the structural integrity of the carrier is fundamental to maintaining a well-defined microenvironment and ensuring stable enzyme performance. However, many confined materials are susceptible to swelling, framework hydrolysis, bond cleavage, or pore collapse under aqueous conditions, high temperatures, or pH variations [79,80,81]. These processes can weaken or completely eliminate the confinement effect. To address these limitations, the current research focus is on strengthening inter-unit connectivity, tuning interfacial interactions, and constructing core–shell structures.

**Figure 4:**
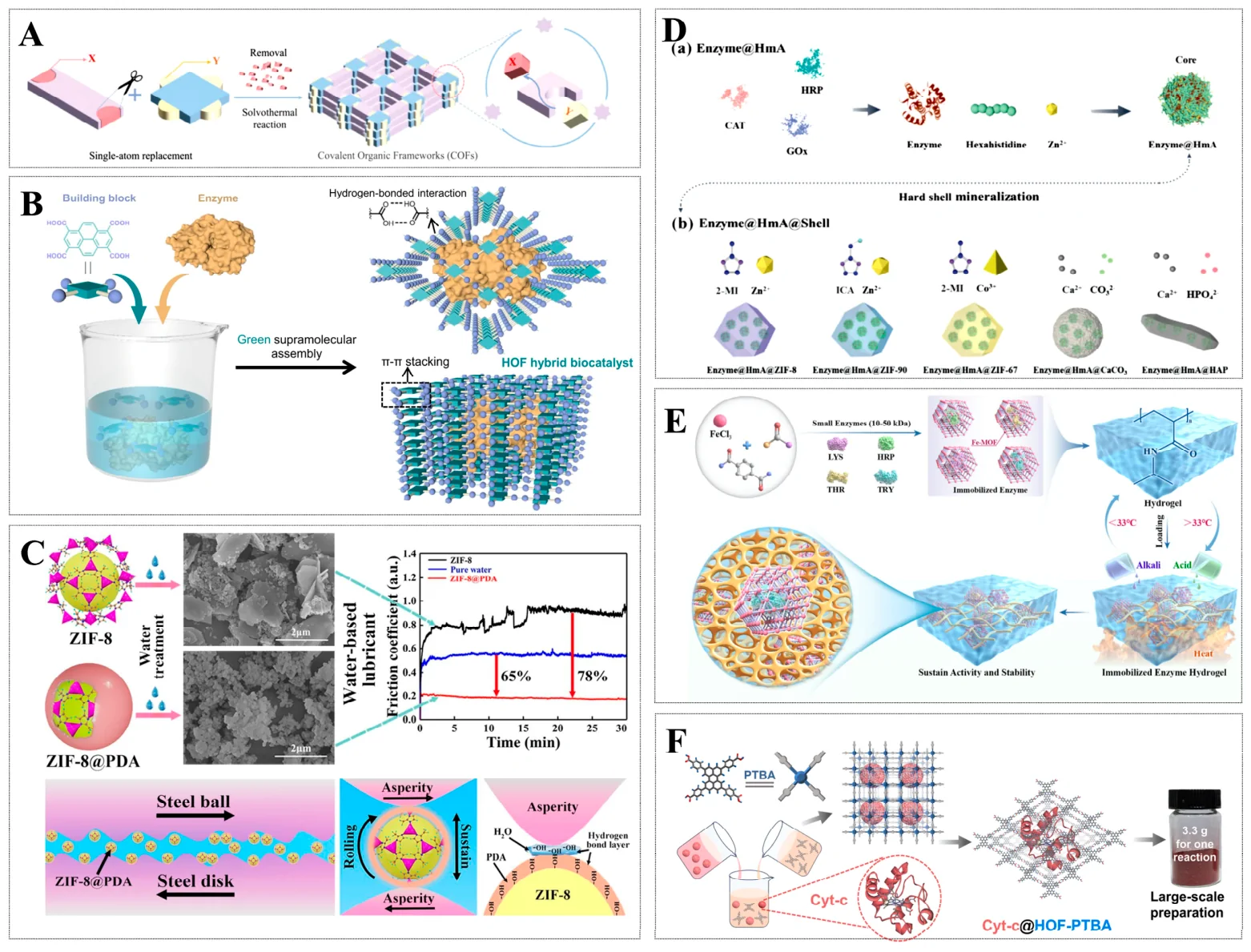
Representative strategies for enhancing the stability of confined enzyme systems. (**A**) Irreversible covalent COF frameworks constructed via Katritzky reactions increase scaffold rigidity. Reprinted with permission from Ref. [83]. Copyright 2025 Wiley. (**B**) Supramolecular assembly enhances HOF stability, improving enzyme stability. Reprinted from Ref. [84]. (**C**) Polydopamine coating mitigates ZIF-8 hydrolysis. Reprinted with permission from Ref. [85]. Copyright 2022 Elsevier B.V. (**D**) Core–shell structures enhance material stability. Reprinted from Ref. [87]. (**E**) In situ enzyme encapsulation combined with hydrogel wrapping reduces enzyme leakage. Reprinted with permission from Ref. [78]. Copyright 2026 Wiley. (**F**) In situ enzyme encapsulation reduces enzyme leakage. Reprinted from Ref. [90].

At the intrinsic structural level, reinforcing the connectivity between building units is an effective approach to improving hydrolytic and thermal stability and preventing structural collapse at the molecular scale [82]. For example, conventional covalent organic frameworks (COFs) constructed via dynamic covalent chemistry often suffered from reversible bond cleavage (e.g., B–O and C=N linkages). By introducing Katritzky-type reactions, irreversible covalent bonds were formed to suppress framework degradation (Figure 4A) [83]. In addition to covalent reinforcement, supramolecular interactions provided an alternative stabilization strategy. A green self-assembly approach based on hydrogen bonding and π–π stacking enabled in situ encapsulation of enzymes within hydrogen-bonded organic frameworks (HOFs), yielding enzyme@HOF hybrid biocatalysts with enhanced structural stability under extreme acidic (pH = 1) and alkaline (pH = 11) conditions (Figure 4B) [84].

Interfacial regulation offers another route to enhancing carrier stability by controlling interactions between the material and the surrounding environment. Two common strategies include hydrophilic polymer coatings and ligand exchange. For example, to reduce hydrolysis of metal–organic frameworks (MOFs), polydopamine (PDA) coatings formed through dopamine self-polymerization served as a stable hydration barrier, wherein hydroxyl groups effectively mitigated hydrolysis (Figure 4C) [85]. The hydrolysis of ZIF-8 was largely attributed to the susceptibility of surface Zn–N coordination bonds to nucleophilic attack by water. To address this, Zhang et al. replaced surface 2-methylimidazole with 5,6-dimethylbenzimidazole (DMBIM), forming a protective surface layer that reduced water accessibility to metal–ligand coordination sites and effectively suppressed hydrolysis [86]. Overall, regulating water access to coordination environments remains a key strategy for improving the aqueous stability of hydrolysis-prone materials.

When intrinsic material stability is limited, the construction of protective shells provides additional reinforcements. Core–shell or multilayered encapsulation architectures create an external physical barrier that isolates the internal confined space from environmental fluctuations. For instance, Wang et al. developed a soft-core@hard-shell architecture in which enzymes were first encapsulated within an enzyme-compatible hexahistidine metal assembly (HmA) to form a soft core, followed by coating with a rigid porous shell to enhance system stability (Figure 4D) [87]. The key principle underlying these approaches is that a sufficiently robust outer confinement layer is required to ensure sustained protection of encapsulated enzymes.

#### 2.2.2. Reducing Enzyme Leakage to Enhance Long-Term Performance

To improve the long-term operational performance of confined enzyme systems, a central challenge is preventing enzyme leakage. The approaches can be typically classified as either post-synthesis loading or in situ encapsulation.

While post-synthesis adsorption is operationally straightforward, enzyme molecules are prone to leaching due to their reliance on weak non-covalent interactions with the carrier. Hierarchical or multi-level pore structures can greatly reduce enzyme leakage, as demonstrated by Jiang et al., who limited uricase loss to 0.45% over five cycles using a MIL-100(Fe)@MOF-Tm MOF-on-MOF structure [88]. Wang et al. showed that a dual-confinement design using an NC-ZIF framework, where an external shell limited diffusion and an internal nanocage immobilized enzymes, retained high GOx/Hemin activity over ten cycles [89]. Similarly, combining physical confinement with hydrophobic interactions, MP-11 encapsulated in Tb-mesoMOF nanocages showed no detectable leakage over seven cycles due to steric restriction and strong hydrophobic anchoring [50].

Compared with post-synthesis loading, in situ encapsulation inherently improves retention by embedding enzymes during carrier formation, avoiding surface adsorption-related leaching and enhancing immobilization efficiency. Green et al. demonstrated this using a room-temperature aqueous-phase COF synthesis, where covalent interactions between enzyme surface groups and COF monomers, combined with physical entrapment during growth, reduced enzyme leakage to below 2.7%, compared to 65% in adsorption systems [28]. Similarly, studies have shown that enzymes encapsulated in rigid Fe-MOF (Figure 4E) and HOF (Figure 4F) can both effectively minimize the loss of small-molecule enzymes [78,90].

### 2.3. Strategies for Enhancing Enzyme Selectivity

Enzymes exhibit high substrate specificity, a property that greatly benefits biosensing applications by improving the resistance of detection systems to interference. However, even naturally selective enzymes may show limited specificity in complex environments, while nanozymes generally suffer from comparatively poor selectivity. To address these limitations, the structural features and chemical functionalities of confined materials can be exploited to improve catalytic selectivity.

#### 2.3.1. Size Classification

**Figure 5:**
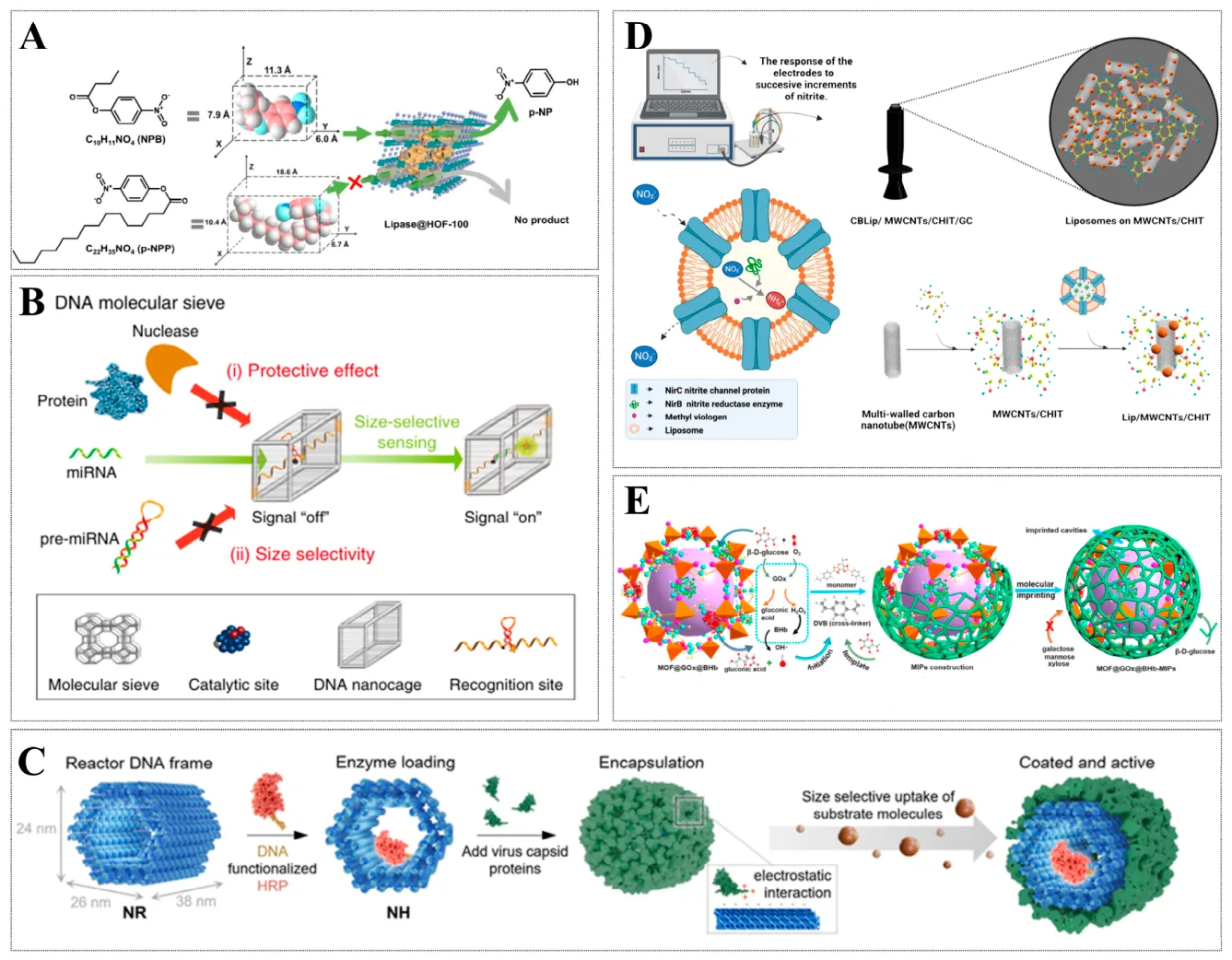
Representative strategies for enhancing the selectivity of confined enzymes. (**A**) HOF-100 pore size as a molecular sieve to enhance enzyme catalytic selectivity. Reprinted from Ref. [84]. (**B**) DNA molecule size as a molecular sieve to improve enzyme selectivity. Reprinted from Ref. [92]. (**C**) Modifiable viral capsid proteins on the enzyme exterior to enhance selectivity. Reprinted from Ref. [94]. (**D**) Confined carriers integrated with substrate-selective channel proteins to increase enzyme selectivity. Reprinted with permission from Ref. [23]. Copyright 2025 Elsevier B.V. (**E**) Molecularly imprinted layers on the enzyme surface to enhance enzyme selectivity. Reprinted with permission from Ref. [104]. Copyright 2021 Elsevier B.V.

Achieving high catalytic selectivity largely depends on controlling substrate access to the enzyme active site. Confined materials with well-defined pore structures provide an effective approach through size-selective molecular sieving. These frameworks create physical barriers that regulate interactions between substrates and catalytic centers [91,92]. For instance, the pore structure of ZIF-8 blocked bulky substrates such as ABTS but allowed smaller molecules, including 2,6-DMP (~3.4 Å), to diffuse through the framework [93]. Similarly, for the small substrate p-nitrophenyl butyrate (NPB, 6.0 Å × 7.9 Å × 11.3 Å), the catalytic kinetics of Lipase@HOF 100 were comparable to those of free lipase. In contrast, for the larger substrate p-nitrophenyl palmitate (p-NPP, 6.7 Å × 10.4 Å × 18.6 Å), the catalytic kinetic curve of Lipase@HOF 100 was nearly flat, whereas free lipase could efficiently hydrolyze p-NPP. This indicated that the pore window of HOF 100 (1.2 nm × 0.8 nm) imposed a strict gating effect on large substrates (Figure 5A) [84]. This size-selective transport mechanism has also inspired the development of DNA molecular sieves for precise molecular recognition. Fu et al. further developed a DNA molecular sieve based on tunable nucleic acid nanocages (3.4–10.5 nm), which selectively excluded large biomolecules while permitting mature miRNAs (19–23 nt) to access encapsulated DNAzymes, thereby enabling discrimination from larger pre-miRNAs (60–70 nt) (Figure 5B) [92].

In some cases, the intrinsic pores of confined structures are excessively large or contain defects, reducing the effectiveness of native size exclusion. Under these conditions, additional screening layers can be introduced to improve selectivity. For example, double-walled DNA origami-confined enzymes allowed substrates such as oPD, TMB, and ABTS to access the catalytic sites with little size discrimination. After the DNA origami structures were coated with viral capsids, the capsid pores selectively permitted the diffusion of small molecules (~337 Da) while restricting larger substrates such as ABTS in a size-dependent manner. Moreover, the selectivity of substrate transport could be tuned by varying the type and amount of capsid proteins (Figure 5C) [94].

For multi-substrate and cascade reactions involving substrates of different sizes, rational pore design can direct reaction pathways by selectively restricting larger molecules while favoring smaller substrates. For instance, Ren et al. employed a MOF (Cu TCA) with 6.5 Å pores to facilitate initial CO_2_ and propargylamine reactions within the pores, followed by a subsequent reaction with external oxidized phosphine, which elevated the yield from 40% to over 90% [95]. This size-matching selective mass transport provides confined catalytic systems with a distinct advantage in complex analytical matrices, including serum and food extracts.

#### 2.3.2. Interface Interaction

Beyond size-selective screening, the selectivity of confined enzymes can be further enhanced by exploiting interfacial interactions between the carrier and either the enzyme or the substrate, thereby markedly improving the anti-interference capability of biosensors. For instance, candida antarctica lipase A (CALA) was physically adsorbed onto the hydrophobic surface of ZIF-8. Hydrophobic interactions between the material and the enzyme substantially enhanced the enantioselectivity of the immobilized enzyme in the asymmetric hydrolysis of carprofen methyl ester, increasing the enantiomeric excess (ee) from 92.61% for the free enzyme to 97.42%, while the reaction yield rose from 59.79% to 90.62% [96]. Similarly, Zhou et al. leveraged material–enzyme interactions in a COF-confined Os nanocluster system. By employing ascorbic acid (AA) to control the (101) crystal facet of the Os nanoclusters and incorporating F-containing functional groups to modulate the electronic states and size of the nanoclusters, they successfully constructed a confined enzymatic system that retained only peroxidase-like activity and reduced background signals over 90% [97].

In addition to material–enzyme interactions, interfacial interactions between the carrier and substrate can also be exploited to enhance the selectivity of confined enzymes. Modification of the confined carrier to tune hydrophilicity/hydrophobicity, hydrogen-bonding networks, and electrostatic properties allows deliberate regulation of the surface chemical environment, effectively excluding interfering species of similar size but differing chemical properties. For example, Xu et al. functionalized MOFs via tannic acid (TA) etching to alter the surface charge state. Negatively charged surfaces selectively enriched positively charged dye molecules such as methylene blue (MB), whereas similarly charged solutes were repelled, demonstrating that charge-selective enrichment combined with nanoscale confinement synergistically enhanced the removal efficiency of charged contaminants [98].

Furthermore, the selectivity of confined enzymes can be significantly enhanced through dual interfacial interactions among the carrier, the enzyme, and the substrate. Specifically, porcine pancreatic lipase (PPL) was co-immobilized with L-proline (Pro) on a microporous MOF (MOF-1,4-NDC(Al)). Pro established hydrogen bonds and electrostatic interactions with the PPL active site via its secondary amine and carboxyl groups, while the spatial configuration of its chiral carbon constrained substrate orientation, thereby generating a chiral microenvironment. This strategy elevated the enantiomeric excess (ee) from 36% to 72%, significantly enhancing enantioselectivity [99].

Overall, non-covalent interfacial interactions, including hydrogen bonding, hydrophobic effects, π–π stacking, electrostatic interactions, and van der Waals forces, constitute the fundamental physicochemical basis for enhancing the selectivity of confined enzymes. Rational exploitation and meticulous design of these interactions enable the construction of artificial confined microenvironments, thereby achieving deliberate and substantial improvement in enzyme selectivity.

#### 2.3.3. Molecular Structure Recognition

Sole reliance on size-selective screening or simple interfacial interactions can partially exclude interfering species. However, when target molecules and interfering components are highly similar in size or chemical properties, such passive screening strategies often fail to achieve high-level selectivity. To overcome this limitation, the incorporation of specific molecular recognition units during the construction of confined carriers [27], or the formation of template-based molecularly imprinted cavities, can establish local microenvironments that are highly complementary to the target molecules.

Spatial integration of the recognition layer with the enzymatic catalytic layer enables the creation of confined catalytic systems endowed with specific recognition functions [100,101]. Aptamers, as synthetic nucleic acid antibodies, can recognize proteins, small molecules, ions, cells, and even viruses with high affinity and specificity, making them ideal recognition elements for biosensing and molecular recognition applications. Based on aptamer-mediated molecular recognition, Liu et al. constructed an HRP-ZIF-8@P1 confined system and imparted selective degradation capability toward bisphenol A (BPA). The catalytic efficiency of HRP-ZIF-8@P1 was 5.6-fold higher than that of unmodified HRP-ZIF-8, and showed negligible degradation of phenol and para-chlorophenol [102]. Similarly, a dual-aptamer electrochemical biosensor based on MIL-53 demonstrated cooperative recognition and enzymatic catalysis, which enabled highly sensitive and selective detection of the SARS-CoV-2 nucleocapsid protein [103].

Substrate-specific channel proteins enable selective and directional transport of substrates or products based on molecular size, charge, and hydrophilicity/hydrophobicity. Direct incorporation of such channel proteins into confined carriers can substantially enhance selective substrate regulation. Esra et al. were the first to incorporate the substrate-specific channel protein NirC into a liposome-based biosensor, enabling highly selective detection of the target molecule. The NirC nitrite channel protein functioned as a selective barrier, allowing NO_2_^−^ to enter the liposomal cavity for the enzymatic reaction while excluding interfering ions. Electrodes containing NirC displayed significantly higher selectivity than those without the channel, highlighting the crucial role of substrate-specific channels in enhancing sensor performance (Figure 5D) [23].

In addition to introducing biological recognition units, template-guided molecular imprinting strategies also provide an effective approach for enhancing selectivity. During synthesis or post-modification, the target molecule serves as a template around which memory cavities are formed via polymerization or self-assembly, creating shape- and functionality-complementary environments for highly specific recognition. For instance, molecularly imprinted nanoreactors with spatial confinement were constructed to detect β-D-glucose selectively in a nanocage cascade enzyme system (Figure 5E) [104]. Similarly, GOx confined on a Ti-NiCo_2_O_4_@Fe_3_O_4_-Au nanoarray was coated with a molecularly imprinted polymer, which permitted β-D-glucose to access the enzyme active center while excluding mannose and xylose, thereby increasing the relative selectivity coefficients over 800% [105]. Moreover, Bose et al. employed micelle hydrophobic cores to create nanoscale confined environments and prepared artificial epoxidases via molecular imprinting. This enabled specific recognition of structurally similar α-olefins such as 1-octene and 1-nonene, achieving atom-level selective epoxidation under mild aqueous conditions [18]. Molecular imprinting precisely controls substrate access and catalysis through specific artificial binding sites, providing an effective strategy to enhance nanoreactor selectivity and guide the design of high-performance biosensing systems.

In summary, the exploitation of material size, chemical properties, and rational modifications can substantially enhance the selectivity of confined systems, enabling precise differentiation between target molecules and interferences and achieving more efficient and controllable performance.

Previous sections have discussed confined enzyme catalysis from three complementary aspects: catalytic enhancement, stability improvement, and selectivity regulation, with different strategies summarized and compared (Table 1). However, these strategies rarely act independently in practical systems, and their performance depends on the integration and balance of multiple mechanisms.

These trade-offs indicate that confined enzyme systems should be optimized as integrated catalytic microenvironments rather than through independent improvement of individual parameters. For example, enhancing stability through stronger confinement may compromise substrate transport, whereas increasing accessibility may weaken enzyme retention. Thus, optimizing a single parameter does not necessarily ensure the best overall catalytic performance; future designs should focus on balancing confinement strength, molecular mobility, and interfacial regulation to maximize the efficiency of confined enzymatic systems.

Combining complementary strategies can generate synergistic effects. For example, integrating structural stabilization with conformational regulation, or combining microenvironment control with cascade organization, can achieve better performance than individual strategies alone. Therefore, rational integration of multiple confinement strategies may provide greater potential for improving confined enzyme systems. However, systematic understanding of the compatibility and synergistic limits among different strategies remains insufficient. Key issues, including the optimal confinement degree, the balance between substrate enrichment and mass transport, and the trade-off between selectivity and activity, require further investigation to establish general design principles. Moreover, the effectiveness of confinement strategies strongly depends on material properties, including pore structure, surface chemistry, and framework rigidity. Therefore, confinement strategy and material selection should be considered together to enable rational design of advanced confined enzyme systems.

## 3. Properties of Enzyme-Confining Materials and Their Applications in Biosensing

**Figure 6:**
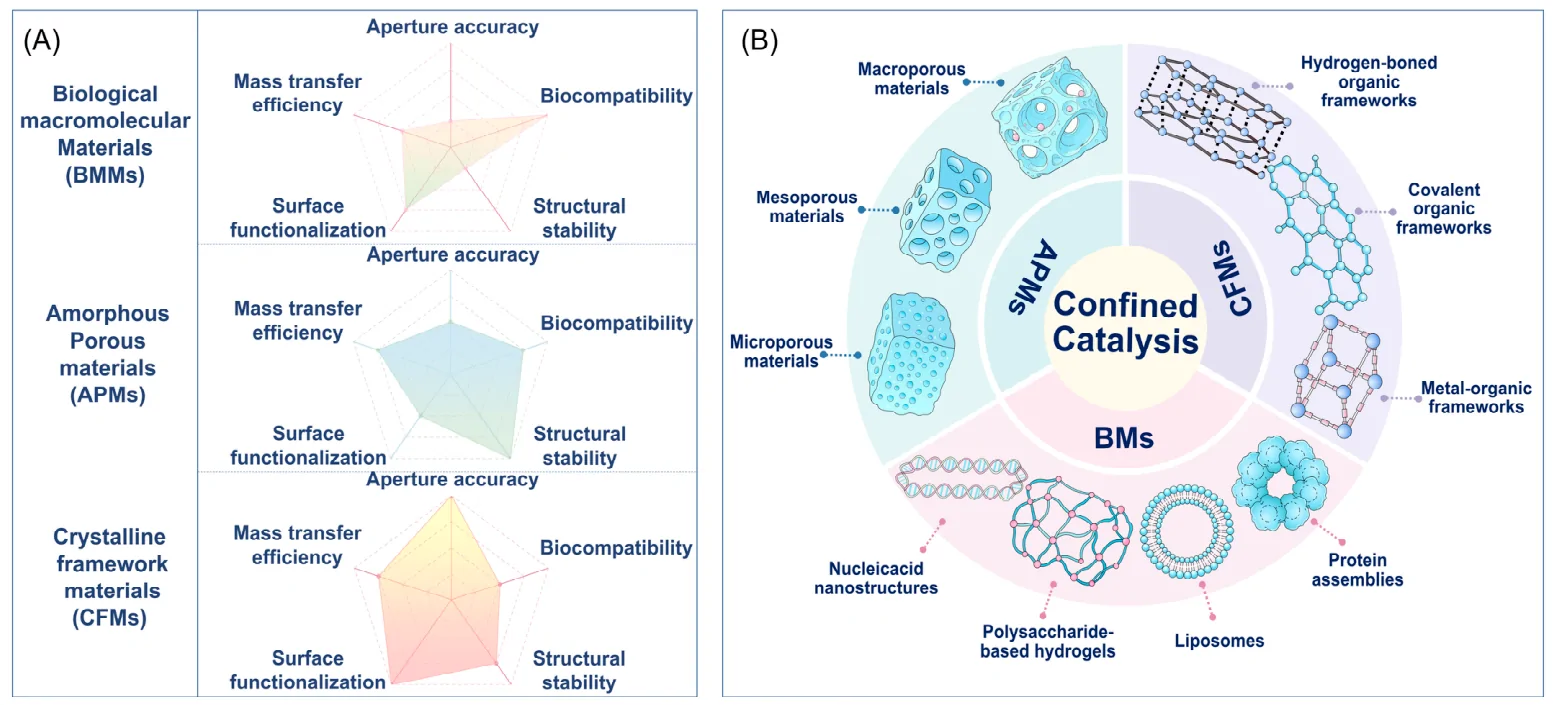
Classification and summary of confinement materials. (**A**) Comparison of material properties. (**B**) Schematic illustration of material structures.

While various enzyme-confinement strategies have been developed to regulate catalytic behavior, their practical performance is largely determined by the physicochemical characteristics of the supporting materials. The carrier not only provides structural confinement but also governs enzyme stability, mass transport, biocompatibility, and long-term operational performance. This section reviews three principal classes of materials commonly employed for enzyme confinement. A summary of their structural features and schematic diagrams is illustrated in Figure 6, and our discussion further elaborates on the characteristics, immobilization mechanisms, and representative biosensing applications of each class.

### 3.1. Biological Macromolecular Materials

Biological macromolecules, including polysaccharides, proteins, lipids, and nucleic acids, constitute a well-studied category of enzyme-confining materials. These molecules can self-assemble into stable architectures via hydrogen bonding, hydrophobic interactions, electrostatic forces, and molecular recognition. Due to their intrinsic biocompatibility and biomimetic characteristics, they generally provide a supportive microenvironment that preserves enzyme conformation and catalytic activity. However, compared with synthetic porous frameworks, biomacromolecular materials generally exhibit lower mechanical robustness and structural reproducibility, which may limit their application in long-term continuous monitoring. Applications range from measuring metabolites like glucose and lactate in blood, sweat, and interstitial fluid to detecting lower-concentration biomarkers in clinical settings.

#### 3.1.1. Polysaccharide-Based Hydrogels

**Figure 7:**
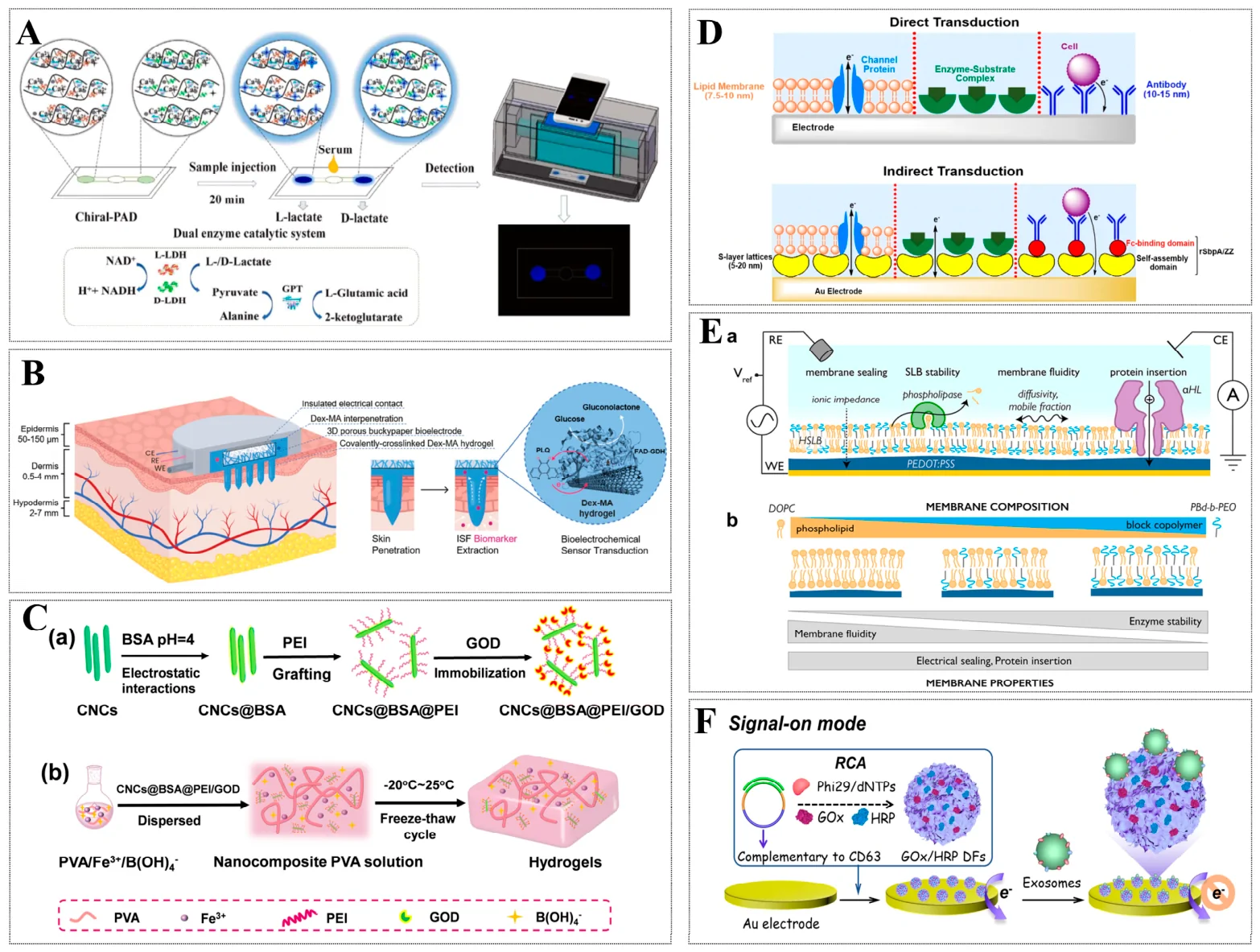
Biosensing Applications of Biomacromolecular Materials. (**A**) Schematic of chiral assays using hydrogel chiral-PAD, with smartphone-based detection and data acquisition. Reprinted from Ref. [108]. (**B**) Integrated biopolymer hydrogel-bioelectroenzymatic sensor: skin penetration, transdermal ISF extraction, and glucose transport from dermis to three-electrode bioelectrocatalytic sensor. Reprinted with Ref. [117]. (**C**) (**a**) Synthesis of CNCs@BSA@PEI/GOD nanomaterials. (**b**) synthesis of nanocomposite hydrogels. Reprinted with permission from Ref. [118]. Copyright 2025 Royal Society of Chemistry. (**D**) Direct (surface-close) vs. indirect (shuttle-mediated) electron transfer: S-layer lattice as intermediate matrix. Lipid-based biosensor uses channel proteins for transmembrane transfer, detection biosensor transfers electrons from enzyme–substrate complex or cell/antibody to electrode. Reprinted from Ref. [124]. (**E**) Polymer-modified hybrid supported lipid bilayer (HSLB) biosensing platform. Reprinted with permission from Ref. [134]. Copyright 2023 American Chemical Society. (**F**) Signal-on electrochemical exosomes aptasensor using CD63-incorporated GOx/HRP DFs as sensing interface and recognition element. Reprinted with permission from Ref. [150]. Copyright 2021 Elsevier B.V.

Polysaccharide hydrogels are widely used as enzyme immobilization matrices because of their biocompatibility, biodegradability, and tunable network structures [106,107]. Their hydrated, three-dimensional porous networks can accommodate biomacromolecules to minimize enzyme denaturation and activity loss (Figure 7A) [108,109]. Hydrogels can be classified according to their mechanical stiffness into three categories: ultra-soft (G′ < 1 kPa), elastic (G′ = 1–100 kPa), and stiff (G′ > 100 kPa). The optimal hydrogel stiffness depends strongly on application requirements. Soft hydrogels favor biological integration and conformational preservation, whereas mechanically reinforced hydrogels are more suitable for wearable devices requiring long-term structural stability. Stiffness, largely determined by crosslinking density and polymer concentration, directly affects enzyme microenvironments, substrate diffusion, and mechanical compatibility with tissues.

Ultra-soft hydrogels with water contents up to 95% closely mimic the mechanical environment of soft tissues and provide enzymes with conformational freedom comparable to that in solution. Their high sensitivity to pH and ionic strength renders them suitable for wearable and implantable biosensors [110]. For example, a redox-responsive supramolecular hydrogel with G′ ≈ 100 Pa was loaded with horseradish peroxidase (HRP) and glucose oxidase (GOx), showing controllable release of fluorescent dyes in response to glucose concentration [111].

Elastic hydrogels combine moderate mechanical strength with efficient substrate diffusion, facilitating applications in flexible electrochemical sensors, microfluidic enzyme reactors, and patch-type sweat sensors. Reactive groups on polysaccharide chains, such as carboxyl, amino, and thiol groups, allow oriented enzyme immobilization, enhanced ion exchange, and controlled degradation [107,112,113,114]. A Ca^2+^-induced alginate hydrogel employing egg-box crosslinking effectively confined lactate dehydrogenase, alanine aminotransferase, cofactors, and substrates while maintaining high mass-transfer efficiency [115]. Glycosyl-based hydrogels are also widely applied in microfluidic biosensing systems. Paper-based microfluidic devices modified with chitosan lowered detection limits for glucose and uric acid to 23 μM and 37 μM, respectively [116]. Importantly, the hydrogel matrix can be formed in situ directly on paper substrates, providing strong adhesion suitable for fabricating portable devices, making it particularly well-suited for point-of-care testing (POCT) platforms.

Stiff hydrogels (G′ > 100 kPa) often require double-network or nanocomposite reinforcement for dimensional stability and wear resistance. Dextran methacrylate (Dex-MA) hydrogels encapsulated FAD-dependent glucose dehydrogenase (FAD-GDH) and redox mediators in microneedle arrays, supporting up to 10 days of continuous glucose monitoring with cell viability > 94.5% after 7 days (Figure 7B) [117]. A composite hydrogel based on cellulose nanocrystals (CNCs), BSA, PEI, and PVA formed a self-healing network with 87.5% efficiency and fracture strength ≈ 0.8 MPa, enabling interference-resistant glucose detection over 1–100 mM (Figure 7C) [118].

Overall, polysaccharide-based hydrogels offer a unique combination of structural adaptability, enzyme stabilization capability, and biological compatibility. Recent developments have further introduced antifouling functionalities [119] and stimulus-responsive behaviors [120], which may mitigate biofouling and enable adaptive sensing under dynamic physiological conditions. Nevertheless, these advanced functionalities remain largely in proof-of-concept studies, and their integration into practical biosensing systems requires further investigation, particularly regarding long-term stability, large-scale fabrication, and clinical translation.

#### 3.1.2. Protein Assemblies

Protein assemblies are constructed based on the diverse reactive groups of amino acid side chains, with assembly processes typically driven by disulfide bonds, hydrogen bonds, and hydrophobic interactions. By mimicking the structural characteristics of natural biological membranes, these assemblies support oriented enzyme immobilization and effective preservation of enzymatic activity [121,122].

These carriers typically exhibit high specific surface areas (50–150 m^2^/g) and excellent biocompatibility, providing optimal microenvironments for enzyme loading, which can reach up to 150 μg enzyme per mg scaffold, while maintaining long-term stability [121,123]. Nevertheless, their structural precision and chemical tunability remain inferior to crystalline porous materials such as MOFs and COFs, limiting precise control over nanoscale catalytic environments. Acting as an interface layer between electrodes and biorecognition elements, S-layer lattices reduce non-specific interactions and facilitate substrate diffusion, enhancing interfacial stability and sensing performance (Figure 7D) [124].

Protein assemblies also enable modular construction of programmable sensing interfaces. Covalent protein-coupling systems, such as SpyTag/SpyCatcher, allow site-specific integration of enzymes with antibodies, aptamers, and other recognition elements under mild conditions [125]. For example, UdgX-assisted covalent coupling enabled aptamer–enzyme conjugates that detected hemoglobin with a limit of detection of 5 nM in serum samples [126]. At the nanoscale, protein scaffolds can further improve catalytic performance through enzyme enrichment and multivalent signal amplification. Virus-like particles (VLPs) provide ordered platforms for assembly immobilization over 200 NanoLuc molecules per particle, achieving ~15-fold signal enhancement and thrombin detection down to 50 pM [127].

Overall, protein-based assemblies combine biocompatibility, structural programmability, and efficient enzyme immobilization. By regulating enzyme orientation, local concentration, and interfacial microenvironments, they provide versatile platforms for biosensing applications. However, challenges related to structural stability, reproducibility, and large-scale fabrication remain before broader practical deployment.

#### 3.1.3. Liposomes

Liposomes are among the most widely studied biomimetic carriers for enzyme confinement. Composed of amphiphilic phospholipids, they spontaneously assemble into bilayer vesicles in aqueous environments, forming membrane architectures that closely resemble those of living cells. This structural similarity provides a biocompatible microenvironment for enzyme immobilization while facilitating integration with biological systems [128,129,130]. Typical liposomes have diameters ranging from 50 to 200 nm and encapsulation efficiencies of 60–85%, depending on the enzyme and lipid composition.

A key advantage of liposomal systems is compartmentalization. Encapsulated enzymes are shielded from external perturbations, enhancing stability and substrate interactions. For example, glucose oxidase confined within 120 nm liposomes retained 72% of its initial activity after 7 days, compared to 40% for free enzyme, and enabled glucose detection over a 0.01–10 mmol/L linear range with a LOD of 1.3 μmol/L [131,132,133]. Supported lipid bilayers provide another strategy. Hybrid supported lipid bilayers (HSLBs) composed of phospholipid-block copolymer mixtures maintained high membrane fluidity across a broad compositional range. They formed stable layers on PEDOT:PSS surfaces, reducing membrane resistance. This structure preserved the native microenvironment of membrane-bound enzymes while improving resistance to degradation, providing a stable and biocompatible platform for enzyme immobilization (Figure 7E) [134].

Beyond enzyme stabilization, lipid-based carriers have frequently been combined with nanomaterials to improve analytical sensitivity. For example, Danni Wang and colleagues constructed a plasmonic colorimetric sensor by integrating gold nanobipyramid etching substrates with HRP-loaded liposomes for signal amplification, enabling sensitive visual detection of telomerase activity for early cancer diagnosis [135]. The phospholipid bilayer also provides interfacial isolation, physically separating catalytic cores from the environment while permitting surface modification with recognition elements [136]. Liposome-based nanoreactors have been used to stabilize single-atom nanozymes, achieving oxidase-like activity that catalyzed substrate conversion with turnover numbers (TON) > 5000 h^−1^ and enabling femtogram-level virus detection [137].

Another feature of liposomes is the tunability of membrane properties. Membrane fluidity, permeability, and stability can be regulated through lipid composition or incorporation of functional components, allowing liposomes to respond to environmental cues such as pH, redox conditions, temperature, and ultrasound [131,138]. For instance, pH-gradient-responsive liposomes allowed controlled pore formation, detecting laccase inhibitors at 6–30 nM in fruit juice with recovery rates of 85–101%, outperforming conventional liquid chromatography [139].

Overall, liposomes integrate compartmentalization, interfacial isolation, and tunable membrane properties. Typical vesicles achieve enzyme encapsulation efficiencies of 60–85%, activity retention over 70%, and LOD improvements by 10–100 times compared with free enzymes, making them highly attractive for biosensing and enzymatic catalysis.

#### 3.1.4. Nucleic Acid Nanostructures

Nucleic acid nanostructures are constructed from DNA or RNA building blocks via programmable base-pairing interactions. By incorporating chemical modifications, such as phosphorothioate linkages, fluorophores, or covalent crosslinkers, researchers can assemble well-defined architectures including tetrahedra, cubes, nanotubes, and nanospheres [140]. Compared with other biomacromolecular matrices, nucleic acid assemblies offer precise control over geometry, size, and surface functionality at the nanometer scale [141,142,143].

The most distinctive advantage of nucleic acid frameworks is their programmability. Similar to writing computer code, the geometry, dimensions, and surface chemistry can be precisely defined. For example, rectangular DNA origami tiles (~60 × 80 nm) have been used to position glucose oxidase (GOx) and horseradish peroxidase (HRP) with inter-enzyme distances ranging from 10 to 65 nm, enabling systematic study of distance-dependent cascade reaction activity [144]. Such spatial control allows modulation of enzyme proximity, local concentration, and assembly configuration, which directly influences cascade efficiency [145,146].

Beyond static encoding, nucleic acid nanostructures can support dynamic functional regulation. DNA nanocages have been designed to confine miR-21-responsive DNAzyme sensors, where complementary base pairing enables logic-gate-like activation and cargo delivery only in the presence of both miR-21 and Zn^2+^ [147]. This highlights the potential for programmable, stimulus-responsive operation in complex biological environments.

The highly defined spatial arrangement of nucleic acid scaffolds also enhances enzyme confinement [148]. RCA-derived scaffolds and flower-like porous frameworks co-localize GOx and HRP, increasing effective local enzyme concentrations, reducing diffusion distances, and maintaining structural stability via Mg^2+^-mediated interactions (Figure 7F) [149,150]. Three-dimensional rigid frameworks further shield enzymes from nucleases and improve cascade kinetics [151,152]. For instance, a near-infrared light-activated DNA nanocage accelerated catalytic hairpin assembly by 10-fold, increased local nucleic acid concentration 33-fold, and achieved a detection limit of 20.81 fM for miRNA-21 in living cells and tumor models [153].

Overall, nucleic acid nanostructures integrate precise spatial programmability, compartmentalized confinement, and biocompatible microenvironments. They allow tailored enzyme immobilization, modulation of inter-enzyme spacing, and protection from environmental interference, thereby enhancing biosensor sensitivity, specificity, and operational stability. These features make them promising platforms for disease diagnostics, environmental monitoring, and single-molecule detection.

Biomacromolecular assembly materials provide biomimetic confinement platforms that combine structural organization with biological functionality. Depending on the building blocks employed, these systems offer complementary advantages, including the molecular recognition capability of proteins, the compartmentalization and interfacial isolation of lipid membranes, and the precise spatial programmability of nucleic acid nanostructures. Such features enable effective regulation of enzyme localization, substrate transport, and catalytic processes, thereby enhancing biosensing performance. However, their practical application is often limited by insufficient structural robustness, as most assemblies rely on relatively weak and reversible non-covalent interactions, which may lead to enzyme leakage and performance degradation. Future efforts should focus on improving assembly stability and reproducibility while maintaining the biocompatibility and dynamic characteristics that underpin their unique advantages.

### 3.2. Amorphous Porous Materials

Amorphous porous materials integrate the structural disorder of amorphous solids with the large interfacial area of porous frameworks. Their short-range atomic order, isotropic coordination environments, abundant dangling bonds, and unsaturated surface sites provide flexible enzyme–support interactions, which are beneficial for improving enzyme loading, interfacial stability, and catalytic accessibility [154,155]. However, these disordered structures also complicate the prediction and rational regulation of enzyme–support interactions, making structure–activity relationships more difficult to establish than in crystalline porous materials. Beyond enzyme immobilization, their porous architectures are particularly advantageous for enzyme-confined biosensors, where spatial confinement and enlarged reaction interfaces help suppress enzyme leaching while improving catalytic efficiency and sensing stability.

The performance of amorphous porous supports is mainly governed by pore size, pore connectivity, accessible surface area, and surface chemistry. These factors determine enzyme retention, substrate diffusion, electron or ion transport, and product release. According to pore diameter, amorphous porous materials are generally divided into microporous materials (<2 nm), mesoporous materials (2–50 nm), and macroporous materials (>50 nm). The following sections discuss these three types of materials with emphasis on their structural features, enzyme-confined mechanisms, and recent applications in biosensing.

#### 3.2.1. Microporous Materials

Microporous materials exhibit high specific surface areas (up to 7000 m^2^/g), abundant active sites, well-defined molecular size selectivity, and strong confinement effects [156,157]. In enzyme-confined biosensing, their main advantage lies in their strong confinement capability. Furthermore, because enzyme molecules are typically larger than the pore dimensions, enzyme immobilization in these systems generally depends on in situ encapsulation, surface anchoring, or hierarchical porous architectures, rather than simple pore filling [158]. Among microporous supports, porous carbon materials and porous polymers are the most frequently used.

**Figure 8:**
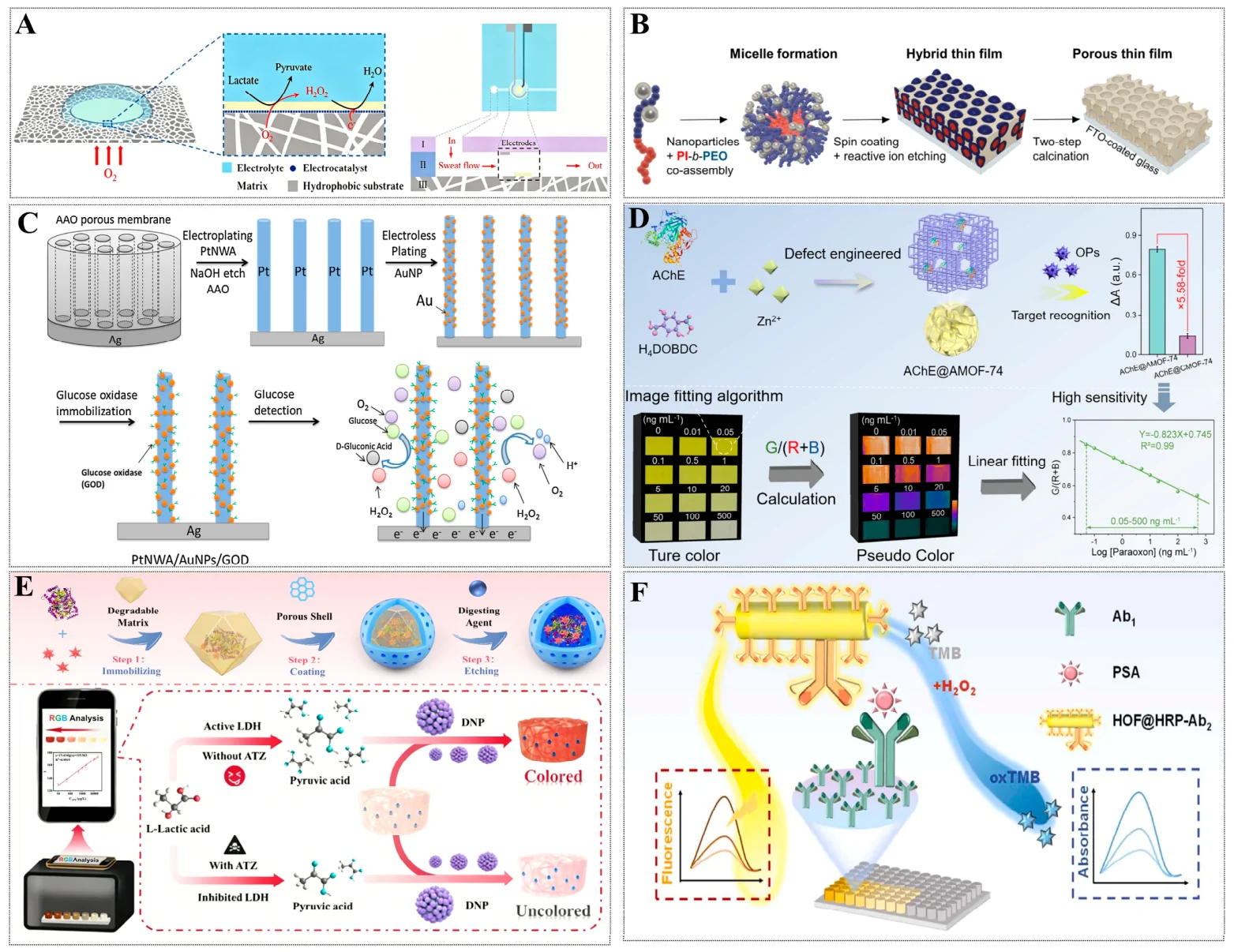
Biosensing Applications of Amorphous porous materials and Crystalline framework materials. (**A**) Schematic diagram of the solid/liquid/gas three-phase enzyme electrode and the cascade bioelectrocatalytic reactions at the local interface microenvironment. Reprinted with permission from Ref. [160]. Copyright 2025 American Chemical Society. (**B**) Schematic of the fabrication process of porous aluminosilicate thin films. Electrochemical detection of glucose and SC2 porous sensor (potential: +0.6 V vs. Ag/AgCl). Reprinted with permission from Ref. [173]. Copyright 2023 American Chemical Society. (**C**) Schematic illustration of the fabrication process for the PtNWA/AuNPs/GOD electrode. Reprinted with permission from Ref. [183]. Copyright 2017 Elsevier B.V. (**D**) The amorphous AChE@AMOF-74-based biosensor for highly sensitive detection of organophosphate pesticides. Reprinted from Ref. [27]. (**E**) Schematic of LDH/NAD+ @COF-42-based visualization sensor for ATZ detection. Reprinted with permission from Ref. [185]. Copyright 2025 Elsevier B.V. (**F**) Schematic demonstration of PSA dual-modal detection. Reprinted with permission from Ref. [206]. Copyright 2024 Elsevier B.V. All permissions for reuse have been obtained via RightsLink or under the respective publishers’ policies.

Porous carbon materials, including activated carbon, carbon molecular sieves, hollow porous carbon spheres, and porous carbon nanotube-based systems, are attractive because of their chemical stability, thermal resistance, relatively low cost, and tunable surface chemistry. Their interconnected porous structures provide large electrochemical interfaces and can facilitate electron and ion transport, which is particularly important for electrochemical biosensors [159]. In practical enzyme-confined systems, restricted substrate diffusion, insufficient oxygen supply and slow electron/ion transport may weaken the apparent catalytic efficiency and long-term stability. Therefore, many studies have focused on engineering the porous interface rather than simply increasing the surface area. For oxidase-based biosensors, porous carbon frameworks can be designed to improve oxygen accessibility and interfacial mass transport. For example, three-phase porous carbon electrodes and porous carbon nanotube-based composite systems have been used to enhance oxidase reaction kinetics, maintain framework permeability, and reduce the leaching of functional particles (Figure 8A) [160,161].

Despite these advantages, strong microporous confinement may also restrict enzyme conformational mobility and reduce catalytic activity. To address this limitation, surface chemistry and framework composition are often adjusted, especially through heteroatom doping. The introduction of nitrogen, boron, sulfur, or phosphorus into carbon frameworks can redistribute electron density, generate defect sites, modify surface polarity, and improve conductivity [159]. Different doped carbon systems improve charge transfer and enzyme–support interactions [162], while also helping to maintain catalytic accessibility within confined porous structures. Functionalized carbon nanotube/polypyrrole interfaces mainly promoted electron and ion transport through electrostatic interactions, giving a glucose sensing sensitivity of 119.74 μA·mM^−1^·cm^−2^ [158]. Phosphorus-doped hierarchical porous carbon microspheres couple micropore formation with improved conductivity, producing a peak current response about 130 times higher than that of bare GCE [163]. In AuNPs/N-doped porous carbon, the combination of conductive porous carbon and metal catalytic sites strengthened peroxidase-like activity and enabled H_2_O_2_ detection with a detection limit of 67 nM [12].

Porous polymers represent another important class of microporous supports for enzyme immobilization. Compared with carbon materials, polymer-based porous structures offer more flexible processability, tunable functional groups, and controllable morphology through self-assembly, phase separation, crosslinking, or template-assisted methods. These features make them suitable for constructing soft, enzyme-compatible microenvironments. For example, Zhu et al. developed a layered porous hydrogel system, AChE-MnO_2_@HPH, which maintained enzymatic activity and stability and achieved a pesticide detection limit of 0.63 ng/mL with storage stability for up to 30 days [164]. Porous polymer films can also be designed with hierarchical pore structures. Guo et al. prepared block copolymer films containing both micro- and nanopores through spinodal decomposition-induced phase separation and applied them to glucose oxidase-based biosensors [165].

#### 3.2.2. Mesoporous Materials

Microporous materials with pore diameters below 2 nm were unable to accommodate large biomolecules such as deoxyribonucleic acid and macromolecular enzymes within their pores [166]. Mesoporous materials therefore provide a more appropriate platform for immobilizing small- to medium-sized enzymes such as proteases, lipases, oxidases, and peroxidases [167,168]. Representative examples include mesoporous silica, particularly MCM-41 and SBA-15, and mesoporous metal oxides such as TiO_2_ and ZrO_2_. Their combination of tunable pores and high specific surface areas, typically 500–2000 m^2^/g, enables high enzyme loading while maintaining sufficient mass transport and catalytic accessibility. Mesoporous materials therefore represent a compromise between enzyme loading and molecular accessibility. However, compared with crystalline frameworks, their relatively broad pore size distributions limit precise regulation of enzyme orientation and substrate selectivity.

Mesoporous silica has been widely used because its ordered hexagonal, cubic, or lamellar pore networks can be adjusted through surfactants, polymers, and swelling agents [169,170]. In addition, surface modification not only makes mesoporous silica an excellent enzyme carrier, but also enables it to serve as a platform for constructing customized microenvironment sensing interfaces, thereby improving enzyme activity retention and sensor sensitivity [170,171,172]. Kato et al. demonstrated that tuning pore size and introducing hydrophobic silane groups enhanced the activity of immobilized enzymes compared with unmodified silica, highlighting the importance of interfacial polarity [166]. An ordered porous aluminosilicate film with ~50 nm pores and ~35 nm pore windows was prepared by block-copolymer templating. After APTES amine functionalization, glucose oxidase was immobilized through electrostatic interactions, giving a glucose biosensor with a sensitivity of 0.52 μA·mM^−1^·cm^−2^ (Figure 8B) [173]. For long-term operation, stronger enzyme–support interactions are often required. Accordingly, Tvorynska et al. covalently immobilized lactate oxidase on SBA-15, obtaining high enzyme loading of 270 μg, 93.8% activity retention after 350 measurements, and 96.9% activity retention after 7 months [174].

Mesoporous metal oxides extend these advantages by combining ordered mesoporosity with crystalline or semicrystalline frameworks. Their low cost, ease of miniaturization, and compatibility with integrated circuit fabrication have supported their use in gas sensors and electrochemical biosensors [175,176]. In enzyme-based sensing, metal oxides can provide additional functions, including thermal regulation, oxygen buffering, direct electron transfer, and electrocatalytic signal amplification. Liu et al. immobilized tyrosinase on phase-change microcapsules coated with a TiO_2_ shell, enabling in situ thermal management under high-temperature conditions [177]. Hybrid oxide systems can further improve sensor robustness. Ispas et al. constructed a glutamate oxidase microelectrode based on a CeO_2_–TiO_2_ nanocomposite. By exploiting the oxygen storage and release capability of nanoceria, the biosensor enabled L-glutamate detection under hypoxic conditions [178].

For metal oxide carriers, the key structural requirement is the simultaneous control of mesoscopic order and framework crystallinity. Luo et al. addressed this issue using a resol-assisted evaporation-induced self-assembly strategy to fabricate highly ordered mesoporous Nb_2_O_5_ microspheres with nanocrystalline frameworks, which were subsequently used for electrochemical H_2_O_2_ sensing [179]. In another example, Qiu et al. prepared hierarchical mesoporous indium tin oxide electrodes by a sol–gel method and directly deposited nitrite reductase NrfA from Shewanella oneidensis MR-1. The interface supported direct electron transfer between enzyme and electrode and showed strong resistance to common anions in aquatic environments [180]. Overall, mesoporous materials offer a practical compromise between enzyme loading, structural accessibility, and interfacial tunability, although further improvements in reproducible fabrication and long-term storage stability remain important for real-sample applications.

#### 3.2.3. Macroporous Materials

Macroporous materials, defined by pore diameters above 50 nm, differ from microporous and mesoporous supports in that their large, interconnected cavities allow relatively unrestricted enzyme diffusion, substrate transport, and product release. These features are particularly valuable when the immobilized large biocatalyst or target analyte involves lipids, starch, nucleic acids, or whole cells. Because macropores impose fewer geometric constraints on protein conformation, they can help preserve native enzyme activity and shorten response times. However, their lower specific surface areas, often below 100 m^2^/g, usually provide fewer anchoring sites than mesoporous carriers. Stable enzyme immobilization therefore often requires surface functionalization, hydrophobic interactions, covalent coupling, or nanoparticle-assisted anchoring.

Anodic aluminum oxide (AAO) is one of the most widely studied macroporous or nanoporous supports because its straight, ordered channels provide defined transport pathways and useful dielectric properties. The coupling between AAO nanochannels and interfacial charge regulation can enhance signal transduction in both electrochemical and optical sensing platforms [181]. Chu et al. developed an ultrasensitive electrochemical sensor based on AAO nanochannels. Hydrophobic modification narrowed the effective channel diameter, modulated interfacial charge density, and produced nonlinear amplification of transmembrane ionic currents, enabling highly sensitive miRNA detection [182]. Li et al. fabricated vertically aligned platinum nanowire arrays in AAO templates, coated them with gold nanoparticles, and immobilized glucose oxidase through EDC/NHS chemistry. The resulting three-dimensional hybrid electrode achieved a sensitivity of 184 μA·mM^−1^·cm^−2^ and a detection limit of 15 μM (Figure 8C) [183].

Macroporous metal structures provide an additional route to signal amplification because they combine open mass-transport channels with high electrical conductivity. In a glutamate biosensor, the synergy between glutamate dehydrogenase and nanoporous gold enabled indirect glutamate quantification by monitoring NADH formation over 50–700 μM, with a sensitivity of 1.95 μA·mM^−1^ and a detection limit of 6.82 μM [184]. Chen et al. designed an integrated nanochannel–electrode chip with nanoporous gold electrodes. Spatial confinement increased urease catalytic efficiency, while volumetric confinement enriched product ions and amplified the output signal. The device detected Salmonella typhimurium within 20 min, with a detection limit of 9 CFU·mL^−1^ [185].

Beyond porous metal structures, porous polymer monoliths integrated into microchips provided a reversible enzyme immobilization environment and maintained catalytic activity through regulation of the local microenvironment, enabling reusable glucose sensing platforms [186]. Engineered microchannel architectures can optimize substrate transport toward confined catalytic sites, thereby enhancing nanozyme catalytic efficiency and sensing sensitivity [187]. In addition to physical confinement, molecular-level regulation of enzyme orientation represents another effective strategy for improving biosensing performance. Oriented enzyme immobilization preserves active-site accessibility, reduces steric hindrance, and facilitates substrate conversion. For example, an oriented acetylcholinesterase microreactor integrated into a microfluidic paper-based analytical device improved enzyme accessibility and operational stability through affinity-mediated directional immobilization, enabling sensitive pesticide detection and repeated use [188].

Recent work has increasingly moved from single-scale macroporous carriers toward hierarchical architectures that combine rapid macropore transport with the high surface area and stronger confinement of mesopores or micropores [189,190]. Zhao et al. prepared macroporous–mesoporous silica microspheres by microfluidics for penicillin G acylase immobilization. The immobilized enzyme reached a specific activity of 1033 U/g, with reduced internal diffusion resistance and good operational stability [156]. This direction addresses the central trade-off in macroporous supports: large pores benefit diffusion and enzyme conformation, whereas smaller secondary pores improve loading and retention. Future designs should also draw on crystalline porous materials, particularly their precise pore functionalization and microenvironment control, while retaining the mechanical robustness and mass-transfer advantages of amorphous macroporous frameworks [173,191].

### 3.3. Crystalline Framework Materials

Crystalline framework materials exhibit a well-balanced performance relative to the previously discussed material classes. Their primary advantages include facile surface functionalization and highly tunable pore sizes, which enable precise construction of enzyme-confined microenvironments and support diverse enzyme activity enhancement strategies. Porous framework materials have been extensively applied in electrochemical sensing and biosensing.

Based on elemental composition, crystalline porous materials can be categorized into purely inorganic frameworks and those incorporating organic components. The former mainly comprises zeolites, whereas the latter includes metal–organic frameworks (MOFs), covalent organic frameworks (COFs), and hydrogen-bonded organic frameworks (HOFs). Given that zeolites have seen limited use in enzyme-based biosensing, this section focuses on MOFs, COFs, and HOFs, highlighting their structural characteristics and recent progress in enzyme-confined biosensing applications.

#### 3.3.1. Metal–Organic Frameworks

MOFs are porous crystalline materials assembled from metal ions or clusters and organic ligands [192]. Their structural robustness, tunable pore architectures, and synthetic versatility make them attractive platforms for enzyme immobilization in electrochemical and optical biosensing. Depending on the framework structure, enzymes can be incorporated through in situ encapsulation, pore confinement, physical adsorption, or layered assembly [11,19,78,192,193].

The immobilization behavior of enzymes is strongly influenced by the coordination chemistry of the framework. ZIFs, constructed from divalent metal ions and imidazolate linkers, exhibit acid-sensitive characteristics that are advantageous for stimuli-responsive sensing [194]. This property has been exploited in ZIF-8/HA hybrid systems for PSA detection, where signal amplification enabled a detection limit of 37.27 fg mL^−1^ [195]. In contrast, carboxylate-based frameworks containing high-valence metal centers such as Zr^4+^ and Cr^3+^ offer greater hydrothermal and chemical stability. Materials from the MIL-101 and UiO families maintain enzymatic activity while preserving structural integrity under demanding conditions [88,196,197]. IRMOFs represent another class of stable hosts. Their Zn_4_O secondary building units and BDC-derived linkers generate highly crystalline frameworks with adjustable porosity and large surface areas [197].

Beyond framework composition, ligand engineering is a means of regulating the enzyme microenvironment. Matching pore dimensions to enzyme size can improve conformational stability and catalytic retention. UiO-66-BA illustrates this effect, where pores of 2.5–6 nm closely accommodate horseradish peroxidase (~4 nm), leading to efficient enzyme stabilization and thiol detection at 1 μM [193]. Increasing structural disorder offers a different route to performance enhancement [198]. Compared with crystalline analogues, defect-engineered amorphous MOFs provide larger pores and greater accessibility, resulting in 3.4-fold higher catalytic activity and 5.6-fold stronger target recognition in AChE@AMOF-74 systems (Figure 8D). Surface functionalization can further tailor local environments for biocatalysis. Hydroxyl-functionalized HP-MOF-OH improved carrier hydrophilicity and preserved the secondary structure of cytochrome c, enabling biosensing at the picomolar level [19,196].

Despite generally lower biocompatibility compared with COFs and HOFs, MOFs provide superior electrical conductivity, enhancing electron transfer at electrode interfaces and improving sensitivity and response speed in electrochemical biosensing [199,200].

#### 3.3.2. Covalent Organic Frameworks

COFs are crystalline porous polymers formed by linking rigid organic building blocks through covalent bonds, creating two- or three-dimensional structures with ordered pore channels [201]. Their architecture can be tailored at the molecular level through modification of building units and framework design, enabling customized sensing platforms.

COFs are highly resistant to chemical and thermal degradation, providing robust protection for encapsulated enzymes [32,202]. Hollow COFs prepared with sacrificial templates preserve enzyme activity over time; COF-42 retained 93.25% of catalytic activity initially and 87.63% after 35 days (Figure 8E) [185]. Mild immobilization strategies, including Pickering emulsion interface assembly and supramolecular self-assembly, maintain enzyme native conformation and enhance stability [29]. Supramolecular networks formed via hydrogen bonding and π–π stacking avoid harsh crosslinking agents, ensuring long-term structural integrity. COF design can be approached hierarchically, encompassing pore–channel engineering, framework construction, and pore–framework synergy. Incorporation of functional groups and metal coordination on pore walls modulates hydrophilicity, hydrogen-bonding networks, and electrostatic interactions. For example, Zhou et al. confined osmium (Os) nanocluster enzymes within magnetic COFs in situ, effectively minimizing nonspecific interference in complex biological matrices and enabling a colorimetric/electrochemical biosensing platform [97].

Framework engineering, through topology customization and surface modifications, improves electrode interfacing and electron transfer, facilitating the development of ultrasensitive biosensors. While electrode-interface regulation, such as incorporating functionalized multi-walled carbon nanotubes, mainly improves electron-transfer kinetics and analytical sensitivity [202], integration of mediators and enzymes within biocompatible COF matrices enhances operational robustness against endogenous interference [203]. In parallel, defect engineering offers a means to regulate the local catalytic environment, as evidenced by a 4.49-fold increase in enzyme activity achieved through controlled defect introduction in imine-linked COFs [32]. Surface biofunctionalization further extends the applicability of COFs to selective biomolecular recognition [100]. Beyond these framework-level modifications, synergistic integration of pore architecture and framework functionality can facilitate spatially organized multienzyme systems. For example, Wang and colleagues used a two-dimensional COF (COFETTA-TPAL) with dual pore sizes (3.06 nm and 0.87 nm) as a carrier. Through pore-size matching and hydrogen-bonding interactions, microperoxidase-11 and glucose oxidase were selectively embedded into large and small pores, respectively, constructing a ratiometric electrochemical biosensor for glucose detection [70].

Unlike MOFs, COFs consist solely of light elements and covalent bonds, avoiding metal-ion toxicity and providing superior biocompatibility. However, their poor electrical conductivity limits direct electrochemical applications [32], necessitating hybridization or complementary conductive strategies.

#### 3.3.3. Hydrogen-Bonded Organic Frameworks

HOFs are constructed from organic or metal–organic units connected via hydrogen bonds, offering mild synthesis conditions, biocompatibility, and reversible structural tunability. Enzyme-induced assembly is a widely adopted approach for HOF-based enzyme confinement, where functional groups on enzyme surfaces attract HOF precursors, enabling in situ encapsulation under mild conditions [204].

Conjugated aromatic building blocks confer intrinsic fluorescence to HOFs, which can be enhanced using aggregation-induced emission (AIE) units [205]. Porosity allows selective guest recognition, facilitating fluorescence-based sensing. For example, HRP encapsulated in pyrene-based HOFs enables dual-mode colorimetric/fluorescence immunodetection of PSA with improved sensitivity (Figure 8F) [206]. To enhance stability, metal centers can be incorporated, providing charge-assisted hydrogen bonds and coordination interactions as structural templates while introducing new functionalities such as electronic, catalytic, and magnetic properties [207]. Examples include Fe-HOFs and Co-HOFs, exhibiting peroxidase-mimicking activity, which effectively replaced natural enzymes and achieved signal amplification [74,208], and PdNPs/GOx@HOF-101 enabled robust photoelectrochemical glucose detection in the temperature range of 30–60 °C and pH 4–8, demonstrating excellent environmental stability [209].

To overcome the poor adaptability of enzyme-induced HOF assembly, selective amidation of carboxyl groups on enzyme surfaces tunes surface charge to enrich ligands, enabling in situ HOF growth and yielding stable HBFs with high activity even under harsh conditions [204]. Carraro and colleagues further developed a magnetically responsive enzyme@HOF biocomposite, which could be precisely immobilized in designated chambers using a permanent magnet, enabling sensitive glucose detection over concentration ranges of 0.25–2 mM and 6–20 mM [210].

Despite their advantages, HOFs face challenges: weak hydrogen bonds and structural flexibility can lead to collapse, and lack of functional sites limits post-functionalization. Nevertheless, their metal-free composition ensures high biocompatibility and self-healing capability, making them promising candidates for flexible in vivo sensing applications [211].

Overall, MOFs, COFs, and HOFs represent three complementary strategies for enzyme confinement. MOFs provide superior structural diversity and electronic functionality but may suffer from metal-associated biocompatibility concerns. COFs offer metal-free and programmable structures but generally require additional strategies to improve conductivity. HOFs provide mild assembly conditions and dynamic adaptability, although their hydrogen-bonded networks often show lower structural robustness. Future framework materials should integrate the advantages of these systems rather than optimize a single property.

Finally, we summarize the representative biosensing applications of enzyme-confined materials discussed above (Table 2). Biomacromolecular materials, owing to their excellent biocompatibility and intrinsic molecular recognition capability, enable highly sensitive detection of metabolites, although their mechanical robustness and long-term operational stability remain to be improved. Amorphous porous materials, characterized by abundant pore structures and adaptable surface chemistries, provide a favorable balance between enzyme loading, mass transport, and structural stability, making them particularly attractive for electrochemical biosensing. Crystalline framework materials, benefiting from well-defined pore architectures and precisely tunable chemical functionalities, offer exceptional control over enzyme confinement and molecular transport, thereby enabling highly sensitive detection of trace biomarkers.

Overall, these three classes of enzyme-confined materials exhibit complementary advantages rather than competing characteristics. Biomacromolecular materials are distinguished by their biocompatibility and dynamic adaptability, amorphous porous materials by their balanced confinement and transport properties, and crystalline framework materials by their structural precision and programmable host–guest interactions. Future research is expected to move beyond single-material optimization toward composite and hierarchically integrated architectures that synergistically combine the strengths of different material platforms. Such designs will facilitate the coordinated optimization of confinement effects, enzymatic activity, molecular transport, and signal transduction, ultimately enabling the development of biosensors with enhanced sensitivity, stability, and practical applicability.

## 4. Conclusions and Future Perspectives

Over the past decade, confined enzyme catalysis has emerged as an effective strategy for improving enzymatic performance. The studies summarized in this review demonstrate that rational confinement enhances catalytic sensitivity, stability, and selectivity through multiple mechanisms, including conformational regulation, substrate enrichment, microenvironmental modulation, and cascade synergy. These performance improvements have been achieved across a wide range of material systems.

Despite these advances, the translation of confined enzyme systems into practical biosensing applications continues to face several challenges. One of the major limitations is the lack of a quantitative understanding of the interplay among pore architecture, surface chemistry, and enzyme kinetics, leaving the design of confined systems largely dependent on empirical approaches. Another important challenge is the compatibility and synergistic boundaries of different confinement strategies. Most existing studies focus on validating individual strategies, whereas the interactions among multiple strategies remain poorly understood. For example, whether conformational regulation and structural reinforcement can simultaneously maximize catalytic activity and stability and whether the optimal spacing between enzymes in cascade systems depends on enzyme identity, remain open questions. These knowledge gaps further complicate the optimization of catalytic efficiency and mass transport, which is particularly critical for real-time and continuous sensing. In addition, reproducible fabrication and long-term structural stability are essential for practical applications but have received considerably less attention than catalytic performance enhancement.

Future research should place greater emphasis on understanding the mechanisms of confined catalysis rather than focusing solely on the development of new confinement materials. Advanced in situ characterization techniques capable of monitoring enzyme conformational changes, substrate and product concentration gradients, and local microenvironmental variations under operating conditions will provide valuable insights into the origin of confinement effects. At the same time, integrating multiscale simulations with high-throughput screening platforms based on microfluidics, automated synthesis, and machine learning will accelerate the exploration of the vast design space defined by material systems, confinement strategies, and enzyme combinations, thereby enabling data-driven rational design of confined enzyme systems. Future studies should also move beyond optimizing individual confinement strategies and instead focus on the rational integration of multiple complementary approaches.

With the continued integration of confined enzyme systems into wearable devices, microfluidic technologies, and point-of-care platforms, enzyme-based biosensors are expected to find broader applications in decentralized healthcare, environmental monitoring, and food safety. Achieving this goal will require not only continued advances in confinement strategies but also further progress in mechanistic understanding, rational multi-strategy design, standardized performance evaluation, and reproducible fabrication. Addressing these challenges systematically will be essential for translating confined enzyme catalysis from laboratory research into reliable biosensing technologies for practical applications.

## Figures and Tables

**Table 1:** Strategies for Enhancing the Catalytic Performance, Stability, and Selectivity of Confined Enzymes.

Overall Objective	Strategy	Mechanism	Applicable Scenarios	Advantages	Limitations
Enhance the Catalytic Performance of Confined Enzymes	Conformational and Orientation Control	Modulating enzyme–carrier interactions to regulate enzyme conformation and orientation	Enzyme activity optimization and immobilization	Enhances intrinsic catalytic activity; improves active-site accessibility	Requires precise control of surface chemistry; risk of enzyme deactivation
	Substrate Enrichment and Accessibility Regulation	Optimizing pore structures and introducing electrostatic/hydrophobic/coordination interactions to enrich substrates	Trace detection and complex sample analysis	Improves local substrate concentration and anti-interference capability	Excessive enrichment may cause nonspecific adsorption; poor pore design limits diffusion
	Microenvironment Regulation and Product Elimination	Regulating the local physicochemical microenvironment (e.g., pH) and promoting removal or transformation of inhibitory products	Long-term sensing and reactions involving H^+^ or H_2_O_2_ generation	Reduces product inhibition; maintains optimal enzymatic conditions	Limited regulation range; dependent on carrier properties and mass transport
	Cascade Synergy	co-localizing multiple enzymes and/or integrating enzyme-mimetic catalytic sites	Multi-step reactions and signal amplification	Enhances reaction efficiency through substrate channeling	Complex assembly; potential incompatibility between enzymes
Enhance the stability of Confined Enzymes	Carrier Stability Enhancement	Strengthening framework interactions or introducing protective layers	Long-term storage and harsh operating conditions	Improves structural robustness and extends operational lifetime.	Excessive stabilization may restrict enzyme flexibility; increases fabrication complexity
	Enzyme Leakage Suppression	Physical confinement through encapsulation or confined hierarchical pore architectures	Reusable sensors and continuous-flow systems	Improves enzyme utilization and reproducibility	Encapsulation may reduce substrate accessibility and enzyme activity
Enhance the Selectivity of Confined Enzymes	Size-selective Transport Regulation	Molecular sieving based on pore size exclusion	Separation of molecules with different sizes	Simple and stable; no additional recognition elements required	Limited discrimination for similarly sized molecules; pore blockage risk
	Interfacial Interaction Regulation	Selective interactions via hydrophobic forces, electrostatics, H-bonding, or π–π interactions	Differentiating molecules with distinct chemical properties	Flexible modification and tunable selectivity	Susceptible to competitive adsorption in complex matrices
	Specific Molecular Recognition Regulation	Specific recognition using aptamers, molecularly imprinted sites, or biological receptors	Detection of structural analogs and complex biomarkers	Highest selectivity and molecular discrimination capability	Complex preparation; possible mass-transfer limitations

**Table 2:** Representative biosensing applications of enzyme-confined materials.

Material Category	Material Class	Detection Target	Detection Method	Key Performance	Ref.
Biological Macromolecular Materials	Polysaccharide-Based Hydrogels	Glucose, uric acid	Colorimetry	LOD: 23 μM (glucose), 37 μM (uric acid); Linear range: 0.1–1.0 mM	[116]
	Polysaccharide-Based Hydrogels	Glucose	Electrochemical	LOD: 0.1 mM; Linear range: 25–50 mM; Stability: 10 days	[117]
	Protein Assemblies	Hemoglobin	Colorimetry	Linear range: 6.3–50 nM	[126]
	Protein Assemblies	Thrombin	Bioluminescence	LOD: 50 pM	[127]
	Liposome	Glucose	Electrochemical	LOD: 1.31 μM; Linear range: 0.01–10 mM; Stability: 72% activity retention after 7 days	[133]
	Nucleic acid nanostructures	miRNA-21	Fluorescence	LOD: 19.56 fM; High specificity toward miRNA-21	[153]
Amorphous porous materials	Microporous materials	Glucose	Electrochemical	LOD: 50 μM; Linear range: 50–700 μM; Sensitivity: 119.74 μA mM^−1^ cm^−2^; Stability: 94.94% activity retained after 21 days	[158]
	Microporous materials	Organophosphorus pesticides	Colorimetry	LOD: 0.63 ng mL^−1^; Linear range: 4–400 ng mL^−1^; Stability: 30 days	[164]
	Mesoporous materials	Lactate	Electrochemical	LOD: 12 μM; Linear range: 40–500 μM; Sensitivity: 0.0047 μA μM^−1^; Stability: >92% activity retained after 7 months and 93.8% after 350 measurements	[174]
	Macroporous materials	Glucose	Electrochemical	LOD: 15 μM; Linear range: 15 μM–2.5 mM; Sensitivity: 184 μA mM^−1^ cm^−2^; Stability: 88% activity retained after 21 days	[183]
	Macroporous materials	Glutamate	Electrochemical	LOD: 6.82 μM; Linear range: 50–700 μM; Sensitivity: 1.95 μA mM^−1^	[184]
Crystalline framework materials	MOF	PSA	Electrochemical	LOD: 37.27 fg mL^−1^; Linear range: 1 × 10^−3^–1 × 10^2^ ng mL^−1^; Stability: RSD < 5.6% after 4 weeks	[195]
	MOF	Organophosphorus pesticides	Colorimetry	LOD: 0.05 ng mL^−1^; Linear range: 0.05–500 ng mL^−1^; Stability: >80% activity retained after 50 days	
	COF	Atrazine	Colorimetry	LOD: 3.47 μg L^−1^; Linear range: 5–20,000 μg L^−1^; Stability: 30 days	[185]
	HOF	PSA	Dual-mode (fluorescence/colorimetric)	LOD: 3.68 pg mL^−1^ (colorimetry), 5.01 pg mL^−1^ (fluorescence); Linear range: 0.01–10 ng mL^−1^ (colorimetry), 0.01–2 ng mL^−1^ (fluorescence)	[206]
	HOF	Glucose	Photoelectrochemical	LOD: 0.76 μM; Linear range: 0.1–10 mM; Stability: stable operation for 2 weeks	[209]
	HOF	Glucose	Colorimetry	Linear range: 0.25–20 mM; Stability: 37% activity retained after 1200 cycles	[210]

## Data Availability

No new data were created or analyzed in this study. Data sharing is not applicable to this article.
